# Human Biomonitoring of Environmental and Occupational Exposures by GC-MS and Gas Sensor Systems: A Systematic Review

**DOI:** 10.3390/ijerph181910236

**Published:** 2021-09-29

**Authors:** Valentina Longo, Angiola Forleo, Lucia Giampetruzzi, Pietro Siciliano, Simonetta Capone

**Affiliations:** Institute for Microelectronics and Microsystems (CNR-IMM), National Research Council of Italy, 73100 Lecce, Italy; angiola.forleo@le.imm.cnr.it (A.F.); lucia.giampetruzzi@le.imm.cnr.it (L.G.); pietro.siciliano@le.imm.cnr.it (P.S.); simonetta.capone@cnr.it (S.C.)

**Keywords:** human biomonitoring, POPs, VOCs, airborne hazardous pollutants, GC/MS, gas sensors

## Abstract

Environmental chemicals and contaminants coming from multiple external sources enter the human body, determining a potential risk for human health. Human biomonitoring (HBM), measuring the concentrations of biomarkers in human specimens, has become an emerging approach for assessing population-wide exposure to hazardous chemicals and health risk through large-scale studies in many countries. However, systematic mapping of HBM studies, including their characteristics, targeted hazardous pollutants, analytical techniques, and sample population (general population and occupationally exposed workers), has not been done so far. We conducted a systematic review of the literature related to airborne hazardous pollutants in biofluids to answer the following questions: Which main chemicals have been included in the literature, which bodily fluids have been used, and what are the main findings? Following PRISMA protocol, we summarized the publications published up to 4 February 2021 of studies based on two methods: gas-chromatography/mass spectrometry (GC/MS) and electronic noses (e-noses). We screened 2606 records and 117 publications were included in the analysis, the most based on GC/MS analysis. The selected HBM studies include measurements of biomarkers in different bodily fluids, such as blood, urine, breast milk, and human semen as well as exhaled air. The papers cover numerous airborne hazardous pollutants that we grouped in chemical classes; a lot of hazardous and noxious compounds, mainly persistent organic pollutants (POPs) and volatile organic compounds (VOCs), have been detected in biological fluids at alarming levels. The scenario that emerged from this survey demonstrates the importance of HBM in human exposure to hazardous pollutants and the need to use it as valid tool in health surveillance. This systematic review represents a starting point for researchers who focus on the world of pollutant biomonitoring in the human body and gives them important insights into how to improve the methods based on GC/MS. Moreover, it makes a first overview of the use of gas sensor array and e-noses in HBM studies.

## 1. Introduction

Environmental pollution caused by human activities is one of the most burning issues that humanity has to face. Among the different kinds of environment pollution (air, water, and land pollution), both indoor and outdoor pollution are leading risk factors for disease burden [1,2]. Although associations between environmental pollution and health outcome are complex, it is now known that exposures to environmental pollution are a major source of health risk throughout the world. Environmental chemicals and contaminants enter the body via various pathways (inhaled air, food, water, dust, and consumer products). The harmful effects of these chemicals on human health depend on their toxicity characteristics and on their persistence in the human body; some chemicals are short-lived whereas others are persistent in the environment, bioaccumulating in people (and/or wildlife) through the multiple pathways of environmental exposure, thus potentially leading to adverse health effects [3]. In a traditional assessment of human exposures to natural and synthetic compounds from the environment, occupation, and lifestyle, harmful chemicals are detected in indoor/outdoor air and the inhaled exposure dose is calculated. This approach has shown limitations.

Currently, human biomonitoring (HBM) is recognized as a more appropriate method. HBM involves measurements of biomarkers in bodily fluids, such as blood, urine, saliva, breast milk, sweat, and human semen, as well as exhaled air and other specimens such as feces, hair, teeth, and nails. 

HBM is therefore a method for assessing human exposure to chemicals or their effects by measuring these chemicals, their metabolites, or reaction products in human specimens, which can be considered the natural complementary method of environment monitoring [4]. In current European strategies, HBM is a pillar for assessing health risk in a more focused way than environmental monitoring since it evaluates the internal dose, achieving an estimate of the biologically active body burden of the chemicals [5]. HBM also provides important information on human contamination at an individual level and contributes to understanding the elicited biochemical or cellular effect whose alteration may lead to adverse health effects, bringing us one step closer to early detection of disease and disease prevention [6].

Exposure to environmental pollution occurs throughout life, from the prenatal period onwards; some researchers are investigating prenatal exposure by analyzing the placenta, an organ from which fundamental information on the state of the mother–fetus couple can be extracted but is usually poorly considered [7]. 

Nowadays there is a greater awareness among citizens and political authorities of the emergency of safeguarding human health from the consequences of pollution [8], due to numerous pollutant–disease correlations that have emerged over the years. Based on this, there is greater sensitivity to the environment–health issue.

HBM programs involving the analysis of different biosamples, and different analysis techniques are multiplying all over the world.

Human biomonitoring has been slowly growing for many years but recently has had a strong acceleration thanks to advances in analytical techniques. 

Among analytical techniques, the gas chromatography-mass spectrometry (GC/MS) method is one of the most used methods to identify and quantify biomarkers of environmental exposure in biological matrices [9]. Clearly, a good extraction and purification method must be combined with an analytical method in relation to the characteristics of targeted compounds. They range from classic liquid–liquid extraction (LLE) to the more recently developed solid-phase microextraction (SPME), passing through solid-phase extraction (SPE). Depending on the type of compound and biological matrix, one technique may be more suitable than the others. For example, the SPME-GC/MS method, which requires rather simple and fast sample preparation, is the elective analytical method in clinical and environmental settings for the biomonitoring of VOCs [10]. As a biological matrix, blood, urine, exhaled air, saliva, human semen, or sweat could be used. Several studies focused on compound segregation compared metabolite patterns in biological fluids from the same individuals [11]. 

The primary objective of this systematic review is to survey the most relevant works published in the past few years on human biomonitoring using gas chromatography (GC) coupled to mass spectrometry (MS) as an analytical method. Our review focuses on the measurement of the main gaseous hazardous substances in human bodily fluids (urine, blood, breast milk, exhaled air, semen), comparing the different extraction methods and biological fluids used. Complementary to the analytical systems based on GC/MS, advanced instrumental systems based on gas sensors, initially developed for air quality, agri-food applications, and diagnostics, begin to be used in HBM programs, providing a modern investigation of human specimens and allowing for cheaper and faster large-scale analyses [12].

Hence, we propose a systematic review of the first works that used gas sensors to analyze the volatile organic compounds (VOCs) in human biofluids, encouraging the application of such devices in human biomonitoring. Currently, our research group is one of the few that has applied gas sensors and sensor array-based devices to VOC analysis in HBM studies. Most of the articles concern gas sensors for the detection of markers in the clinical field for applications in diagnostics and prognostics or for monitoring indoor and outdoor air quality. Since several gas sensors detect volatile compounds that are important biomarkers of exposure to pollutants (such as formaldehyde, benzene, toluene), we selected publications related to gas sensors and/or gas sensor-based devices that detect volatile pollutants in biological fluids, though in the reported works they are used for other applications.

## 2. Methods

A systematic review of the literature regarding biomonitoring of pollutants by gas chromatography/mass spectrometry (GC/MS) and/or gas sensors was conducted via the analysis of all scientific publications that dealt with studies on populations exposed to occupational and environmental pollution in which at least one of those two analytic systems was used. 

The literature search was performed on 4 February 2021 according to the Preferred Reporting Items for Systematic Reviews and Meta-Analyses (PRISMA) methodology [13] in three different databases (PubMed, Scopus, and Web of Science) from coverage inception to 2021 (1971 for PubMed, 1788 for Scopus, 1900 for Web of Science); the scientific articles published up to the aforementioned dates and satisfying the eligibility criteria were selected. 

The search strategy consisted of filtering the publications with a combination of keywords specifying the following search strings: “gas chromatography,” “GC-MS,” and “gas sensor” combined with “VOC” and/or “environment pollution” and with different biological fluid names, such as “urine,” “blood,” “breath,” and “sperm.” We also performed an additional manual search by analyzing the reference list of original publications and reviews on the same topics. We transferred the results from databases to a Microsoft Excel spreadsheet where inclusion and exclusion criteria were recorded. The publications were evaluated independently by three reviewers and cases of disagreement were resolved by a fourth reviewer. 

Following PRISMA protocol, the papers were first screened for title and next for abstract. In both steps, according to the exclusion criteria, we excluded studies focused on (1) animals and/or plants, (2) environmental matrices (e.g., water, air, food, soil), (3) diseases (mostly bacterial infections, inflammation, and cancers) without a direct correlation with a pollutant, (4) prenatal stage, (5) endogenous VOCs, (6) in vitro and cellular studies, (7) drugs, and (8) use of different types of detector (FID or ECD), inferable from title or abstract. 

Eligibility criteria were used to examine the publications that passed the first two steps and to select the articles to be included in this review. The studies were included if they met the following criteria:

1. In English;
2. Population and non-methodological studies;
3. Fully available;
4. Protocol based on the use of GC-MS or a gas sensor.

## 3. Results

**Figure 1:**
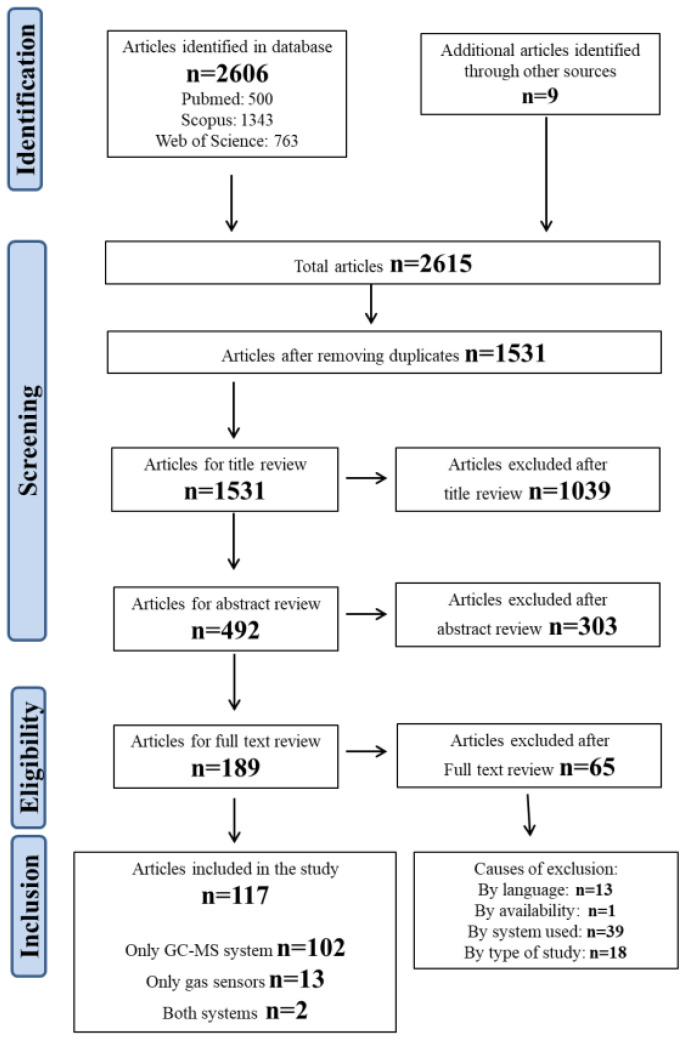
Flowchart of the study selection process.

The search strategy identified a total of 2606 publications, specifically 500 from PubMed, 1343 from Scopus, and 763 from Web of Science. After removing duplicates, the articles to be screened were reduced to 1531. These papers underwent title and abstract review to evaluate their inclusion in the systematic review. Only 189 passed this step and finally those 189 papers were read and reviewed, and of those only 117 were used for analysis, as they meet the inclusion criteria. N = 13 papers were excluded because they were not written in English (n = 3 in French, n = 3 in Japanese, n = 3 in Chinese, n = 3 in Italian, and n = 1 in German), n = 13 because they were focused on the method or its improvement, n = 39 because they involved the use of a detector other than mass spectrometry (such as a flame ionization detector (FID) or electron capture detector (ECD)) and n = 1 because it was not available. The PRISMA flow chart is reported in Figure 1.

Approximately 88% of the selected publications used the GC-MS method (104), whereas only 15 articles involve the use of gas sensors. In only two of our previous works (which are indicated in Figure 1 as “both systems”) were both methods used in comparison with each other. 

The 104 papers (Supplementary Tables S1–S3) covered a wide range of polluting substances (brominated flame retardants (BFRs) (n = 2), benzene (n = 4), phthalates (in particular bisphenol A) (n = 2), benzene–toluene–ethylbenzene–xylene (BTEX) (n = 5), organochlorine pesticides (OCPs) (n = 11), polycyclic aromatic hydrocarbons (PAHs) (n = 14), polybrominated diphenyl ethers (PBDEs) (n = 11), polychlorinated biphenyls (PCBs) (n = 7), polychlorinated dibenzo-p-dioxins and dibenzofurans (PCDDs/Fs) (n = 5), volatile organic compounds (VOCs) (n = 16), and others). The selected publications concerned studies carried out on sample populations in different countries all over the world: USA (n = 1), China (n = 16), Italy (n = 11), Germany (n = 7), Sweden (n = 6), Japan (n = 5), Korea (n = 5), Iran (n = 4), Spain (n = 4), Brazil (n = 3), Finland (n = 2), France (n = 2), India (n = 2), Russia (n = 2), and the remaining 13 studies in Bolivia, Canada, Czech Republic, Ghana, Greenland, Poland, Ukraine, Jordan, Lebanon, Mexico, Nigeria, Pakistan, Romania, Slovenia, and Taiwan. 

**Figure 2:**
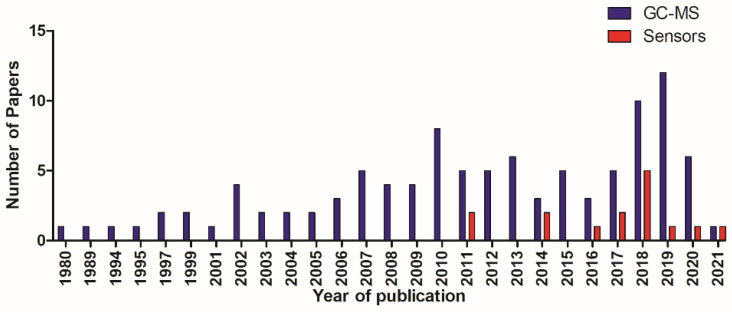
Total number of papers in relation to year of publication.

All articles were published between 1980 and 2021 (Figure 2). 

**Figure 3:**
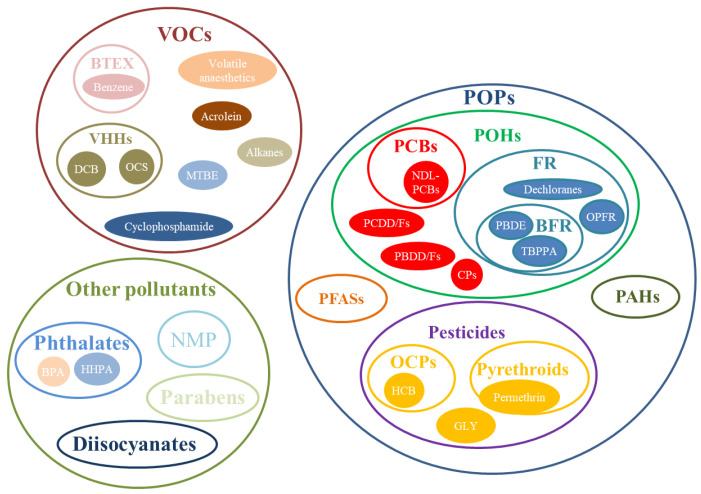
Classification of airborne hazardous pollutants.

The pollutants considered in the selected papers were classified into three main groups (Figure 3).

The interest in health risk assessment in the population was mainly aimed at workers due to the intensity and/or duration of exposure to noxious chemicals, but in recent years, growing attention has been devoted to HBM studies in the general population, as pollutants and harmful chemicals are ubiquitous. Hazardous compounds occur in air, water, food, and a wide range of household and industrial products. Human exposure to contaminants is pervasive and the health risk due to environment pollution affects the entire population. In our review, we highlighted the works that concerned the overall exposure as well as the articles involving occupational exposure, and a particular concern for the most vulnerable population segments emerged. The scientific publications were therefore divided according to the type of exposure into “occupationally exposed workers” (n = 41) and “general population” (n = 63). Among the studies on the general population, some concerned specific categories such as children (n = 13) and women (n = 13). In particular, for women, different stages of life and development were considered: state of pregnancy (n = 3), young mothers (n = 4), lactating mothers (n = 2), post-menopausal women (n = 1), and women with breast cancer (n = 1). In three articles (n = 3), infertility problems were evaluated in relation to different pollutants. 

Among occupationally exposed workers, most investigated job categories were coke-oven workers (n = 5), farmers (n = 2), firefighters (n = 3), and waste-dismantling workers (n = 5).

Another parameter considered for classification in the review was the biological fluid used in the HBM studies. Considering that some articles presented data involving different biological matrices, we included n = 61 papers in which blood samples were analyzed (whole blood (n = 27), blood serum (n = 22), blood plasma (n = 9), and umbilical cord blood (n = 3)). Other biofluids considered in the analyzed publications were urine (n = 41), exhaled air (n = 12), breast milk (n = 8), and human semen (n = 4).

In contrast to the major publications based on GC/MS, only a few publications were based on gas sensors, as HBM is a challenging but relatively new application field for gas-sensing systems. The 15 papers concerning the use of a gas sensors/gas sensor array for the detection of VOCs are listed in Supplementary Table S4. 

## 4. Discussion

The search for scientific publications related to HBM with GC/MS and gas sensors yielded many more publications than expected, mostly based on GC/MS, with only a minority focused on gas sensors. To simplify the discussion of the main outcomes of the systematic review, this section is divided into three complementary subsections following the classification of the airborne hazardous pollutants in the three main groups (POPs, VOCs, other pollutants) shown in Figure 3; gas sensors were discussed as a subsection of VOCs. 

### 4.1. POPs: Persistent Organic Pollutants

Persistent organic pollutants (POPs) are chemical substances that share the combination of two specific characteristics: persistence and bioaccumulation. Numerous chemical compounds have these characteristics, so the POP group is a miscellaneous group of many different pollutant substances. 

In the studies on POPs, some authors preferred to identify as many POPs as possible in the human body, and others focused on a specific class of pollutants. 

In three articles, the authors got a total overview of all POPs present in the blood samples of three different types of population: the general Romanian population [14], Brazilian pregnant women [15], and Canadian children [16]. Despite the different experimental approaches, all works converged on the same principal result: the bodily burden of persistent organic pollutants increases with age because of their characteristic of bioaccumulation. One finding that apparently disagrees with this result was the higher concentrations of polybrominated diphenyl ethers (PBDEs) in Canadian children than in adults. The authors assumed that this experimental result was due to dermal contact and dust ingestion by children and, possibly, to placental transfer and breastfeeding [14]. This result was also reported by Schettgen et al. [17] in German children, supporting the hypothesis that the cause is related to the breastfeeding of babies. In Jogsten et al.’s assessment, PBDE levels vary with no signs of age or gender dependence [18]. 

Furthermore, due to the extensive agricultural activities, pesticide levels were found to be higher in rural areas than in urban ones [15].

Many research groups studied the phenolic organohalogens subgroup, which mainly includes polychlorinated biphenyls (PCBs), polybrominated dibenzodioxins/furans (PBDDs/Fs), polychlorinated dibenzodioxins/furans (PCDDs/Fs), and polybrominated diphenyl ethers (PBDEs). In these papers, the most used biological fluids were blood and breast milk, and the main extraction methods were liquid–liquid extraction (LLE) and/or solid phase extraction (SPE). 

All the authors of the studies on exposure to PBCs agreed that concentrations of PCBs in biological matrices increase in relation to age, geographic area, agricultural practices, and lifestyle [17,19,20,21,22,23]. The most alarming aspect of the data that emerged is that the potential health risk in areas with large industrial settlements and in contaminated sites involves the entire population of residents and workers through multiple pathways. As example, in Parera et al.’s paper [24], the authors did not detect differences in blood POP levels between subjects considered to be exposed and non-exposed to a solid waste incineration (SWI) plant, i.e., there was no relation to the closeness to the SWI. The authors attributed the main route of contamination of these compounds in humans to diet, which is widely accepted to be the major pathway for human dioxin exposure. Moon et al. also got the same result; they did indeed find levels of PCDDs/Fs as well as of PCDD/Fs in the blood of workers at a municipal SWI comparable with those of nearby residents [25]. 

Moreover, Parera et al. [24] interestingly found differences in relation to sex and age; concentrations of PCDD/Fs and PCBs were higher in women than in men, and in the oldest age group. This is consistent with the fact that these substances are fat-soluble, accumulating in adipose tissue over time, and there is a greater proportion of adipose tissue in women or a higher consumption of these compounds in their diets. 

HBM can provide useful quantitative information regarding the actual exposure of a population to environmental pollutants (as POPs), allowing a direct and more precise assessment of the risk distribution in a population even in relation to specific pathologies. 

As examples related to the complex health–environment issue, two important papers demonstrated the direct correlation between the presence of PCBs and a pathological effect. In the case-control study by Charlier et al. [26], the role of PCB153 as a significant risk factor of breast cancer was confirmed, whereas in Kobayashi et al.’s paper [27] highlighted the link between the presence of non-dioxin-like PCBs in maternal blood during pregnancy and the increased H19 and LINE-1 methylation levels in cord blood is highlighted, which implies a reduced birth size, particularly in female infants.

Flame retardants (FRs), a widely studied group of compounds that are integrated into potentially flammable materials (electronic devices, upholstery, textiles) to prevent fire hazard, also belong to the set of phenolic compounds (POHs) [28]. FRs can contaminate air, water, and soil during manufacture or uncontrolled burning and dismantling of electronic and electric waste; chemicals can leak from products into dust and into the air, and dust can get on hands and food and then into the mouth when food is eaten. The potential adverse health effects associated with FRs include endocrine and thyroid disruption, immune system dysregulation, reproductive toxicity, cancer, abnormal development of fetuses and children, and neurological function alteration. Most of these compounds contain one or more bromine atoms, so they are called brominated flame retardants (BFRs). The most dangerous BFRs for safety issues are polybrominated diphenyl ethers (PBDEs), hexabromocyclododecanes (HBCDs), and tetrabromobisphenol A (TBBPA) [28]. In particular, among these, the most observed in human serum are PBDEs [28,29]. 

Many research groups focused on PBDE detection both in the general population and in occupationally exposed workers. Based on extraction methods, these works can be divided in two categories: those that used liquid–liquid extraction (LLE) (mainly with hexane/methyl tert-butyl ether 1:1, v/v) and those that used solid phase extraction (SPE). 

In the articles targeted toward occupational exposure, LLE was always used. Since PBDEs are a class of additive flame retardants widely used in electronic appliances and electrical goods, all these works focused on electronic waste-dismantling workers and/or computer technicians. All the research groups agreed that blood concentrations of PDBEs in electronic waste-dismantling workers, who internalize them via inhalation of airborne particulate, were higher than in the control groups, especially for PBDE-209 [30,31,32]. In other exposed categories such as computer clerks, the PDBE concentration was correlated with the duration of employment of the computer workers [32]. 

Furthermore, these compounds can be oxidized by cell metabolism and accumulated in hydroxylated form (OH-PBDEs), which were found to be correlated with pathological alterations in in vitro and in vivo studies [33].

PBDEs and their metabolites were also detected in the general population. In general, the most used biological matrix was blood. Xu et al. monitored PBDE concentrations in 25 Chinese adults in Shanghai, and they revealed levels comparable to those of people living in other cities and to those of animals, with no correlations with age, sex, or workplace [34]. Alarmingly, these levels were higher in children. 

In a study in the USA, the authors found PDBE concentrations in children two to four times higher than those in adults. In particular, PDBE ingestion increased in relation with hand-to-mouth activity, dietary preferences, and exposures to breast milk [35]. The presence of this class of pollutants in breast milk was confirmed by Inoue et al., who compared PBDE concentration in the blood and breast milk of 89 Japanese women, finding BDE-209 at the highest concentration in serum, and BDE-47 and BDE-153 in milk in all the geographical areas considered in the study [23]. 

Aiming to explore potential contamination in the prenatal period, two projects began to assess PDBE presence in umbilical cord blood. In the first, researchers compared cord blood and breast milk levels obtained from the Korean population: BDE-47 was the most dominant congener both in cord blood and breast milk, whereas BDE-100, -99, and -153 were also found in high concentrations in both biological samples with slight differences. The most important finding was that cord blood samples displayed positive correlation with environmental surrounding factors (oil paint or insecticides), whereas breast milk samples displayed positive correlation with dietary intake (frequency of consuming fish or tea), proving an unlikely source of contamination [36]. The second project was much more recent and extensive, as it concerned 318 Chinese mother–child pairs, from which 318 cord blood samples were obtained. In general, BDE-209 was the most abundant congener, but worrying data showed that cord serum BDE-153 and BDE-154 concentrations were negatively associated with body mass index (BMI) score and waist circumference measured at 7 years of age, with sex-specific effects (mainly in male children), altering child growth [37]. The precise mechanisms on the obesogenic effects of prenatal and postnatal PBDE exposures have not been fully illustrated, but it is supposed that disruption of the thyroid hormone could also be involved in the molecular mechanism, as documented by Kim et al. [36]. 

Since in rodents a neurodevelopmental toxicity related to prenatal exposure to PBDEs has been demonstrated, and based on the experimental evidence given by HBM studies of PBDE exposure even in fetuses and infants both via placenta and breastfeeding, an American research group investigated a hypothetic relationship between exposure to these pollutants and autism. Fortunately, despite PBDEs being very high in all children, no association between children’s circulating levels of PBDEs and autism cases was found in this study [38]. 

In only one study among those involving exposure to PBDEs were urine samples used in addition to blood samples. A correlation between blood PBDE and urinary metabolites was found to exist, both in concentrations and in congener composition [39]. 

Jakobsson et al. demonstrated a clear dose (duration of computer work)–response relationship among computer technicians for some higher brominated PBDE congeners [30]. Moreover, they found another flame retardant, i.e., tetrabromobisphenol A (TBBPA), in the occupationally exposed population and computer technicians and workers dismantling electronics at comparable levels, in accordance with the shorter half-life of TBBPA (approximately 2 days) limiting any human bioaccumulation. 

In 2014 and in 2020, Fromme, along with two different research groups, investigated the concentrations of two types of FRs in the German population. In 2014, he reported a correlation between organophosphate flame retardants (OPFRs) and plasticizers present in dust and in indoor air with some urinary metabolites in German children [40], and in 2020, he found blood levels of dechloranes in German adolescents similar to those observed in 2017, but higher compared to those reported in other European countries [41]. 

Another class of important and very dangerous phenolic organohalogens are polychlorinated dibenzodioxins/furans (PCDDs/Fs). Since 1997, the research group of Hansson [42] declared that PCDDs and PCDFs could be used as markers of occupational exposure and indicated a pattern of PCDDs/Fs as a specific marker for occupational exposure in the polyvinylchloride (PVC) and chloralkali industry.

Looking at a geographical map of PCDD/F exposure risk in Europe, we have two different scenarios: Whereas PCDD/F body burden values in the Belgian population were higher than in neighboring countries [22], in Spain PCDD/F levels decreased in the general population from 1999 to 2002 and stabilized thereafter [24].

Aiming to assess the supposed contribution of PCDDs/Fs to male infertility, Galimova et al. used human semen for the first time for their biomonitoring study, preferring it to blood. [43]. They referred to increased levels of PCDDs/Fs in the ejaculate of infertile males compared to fertile donors and identified a typical profile of PCDDs/Fs corresponding to contemporary industrial manufacture that confirmed the technogenic nature of sperm contamination.

Chlorophenols (CPs) are another kind of POP widely used in pesticides, herbicides, and disinfectants whose mechanisms of pathogenicity to humans have to be better understood. In one study, CPs were detected in the urine of sawmill workers using the GC/MS system; the concentrations of CPs in those workers were comparable to those of the general population and to those of workers in other sawmills, as reported in other studies [41,44]. 

Polycyclic aromatic hydrocarbons (PAHs) are a ubiquitous group of several hundreds of permanent organic pollutants. They include two or more fused benzene rings generated during incomplete combustion of organic matter. They occur naturally in coal, crude oil, and gasoline, and they are also produced by anthropogenic activities (burning of fossil fuels, industrial processes, and automobile emissions, among others). Exposure to PAHs is a primary human health risk for cancer, but it has also been linked with cardiovascular disease and poor fetal development.

Among the 14 papers reported in this review and regarding PAH biomonitoring, nine concerned occupational exposure. Coke-oven workers are the most studied work category for biomonitoring exposure to PAHs: Here, we cited six papers in which this exposure was measured by a GC/MS system using urine samples. The principal extraction method is LLE with hexane, but due to the volatility of 2-, 3-, and 4-ring PAHs, headspace solid phase microextraction (HS-SPME) and thermal desorption were also used. 

Naphthalene (NAP) and phenanthrene (PHE) are two PAHs that were detected both in coke-oven workers and in control groups [45,46], but in general the related urinary concentration was higher in occupationally exposed subjects [47]. Furthermore, benz[a]anthracene (BaA) and chrysene (CHR) were quantifiable only in samples from coke-oven workers. In these papers, exposure to asphalt roofs was also evaluated; the urinary concentration of these PAHs in asphalt workers and road construction workers was in the same range of that found in the not occupationally exposed group [44], a result in line with the worrying common exposure to such environmental pollutants in both living and working environments.

Moreover, the exposure to PAHs of coke-oven workers was strongly associated with pathological conditions such as diabetes, especially in subjects who were also smokers, but it was also associated with overweight condition, longer working years and works in coke-oven settings [48], and impaired heart function. Indeed, the presence of urinary 2-hydroxynaphthalene (2-OH-NAP), following occupational exposure to coke-oven emissions, was associated with a dose-response decrease in heart-rate variability, in relation to working years and exposure levels [49]; urinary 1-hydroxynaphthalene (1-OH-NAP), 2-hydroxynaphthalene, and total PAH metabolites were also associated with increased risk of atherosclerotic cardiovascular disease [50].

The de León-Martínez research group compared four categories of workers highly exposed to PAHs: brickmakers, stonemasons, mercury miners, and indigenous workers. Also in these categories of workers, 2-OH-NAP and 1-OH-NAP were the most present, along with 1-hydroxypyrene (1-OH-PYR), 9-hydroxyfluorene (9-OH-FLU), and 4-hydroxyphenanthrene (4-OH-PHE). PAH fingerprints among the brickmakers and mercury mining population were very similar, whereas the indigenous population presented a different fingerprint compared to the other three groups of workers [51]. 

High levels of PAHs were also found in firefighters [52] and in air force personnel [53], in particular after post-fire suppression activity and work shifts with exposure to fuel substitutes, such as the debated JP8 jet fuel. 

Among non-occupationally exposed subjects, PHE was the most frequently detected substance in blood, whereas NAP was the PAH detected with the highest serum concentration [14,54]. The dominant PAHs in human whole blood were low-molecular-weight PAHs (2–4 rings), whereas higher-molecular-weight PAHs were found at relatively lower concentrations [54]. In general, PAH concentrations were lower in blood than in urine, where BaA and carcinogenic PAHs were more abundant [55]. In a study by Boada et al., serum PAHs did not appear to be related to bladder cancer risk: The only difference between cancer patients and the controls was that pyrene was detected only in the controls and CHR only in the cancer group [56]. 

Only in one study was PAH concentration evaluated in the breast milk of lactating women residing in Lanzhou, a petrochemical industrialized valley in China. A significant correlation was found between human milk PAHs (mainly 3–4 ring PAHs) and the living environment in the different Lanzhou districts. Other factors related to PAH exposure seemed to be barbecue food and secondhand smoke, with a predominance of ingestion intake [57].

Among POPs, another wide class of compounds is represented by pesticides, in particular organochlorine pesticides (OCPs) and pyrethroids. Two research groups that biomonitored blood samples focused on the exposure to pesticides in agriculture farmers and people living within a 1 km range of pesticide-treated fields. In both works, the residues of pesticides in blood were low because those compounds are immediately metabolized in the body, but they could produce negative alterations of body homeostasis such as cholinesterase inhibition [58] and increased oxidative stress inside the body [59]. 

Two other groups investigated the exposure of the general US population to pesticides by analyzing urine compositions. The first detected high levels of organochlorine (OCPs) and organophosphate pesticides (OPPs) in urine samples of pregnant women in Salinas Valley, a highly agricultural area in California, with even higher peaks for several pesticides in women living in other areas characterized by the use of pesticides in agriculture [60]. The second group monitored the Ohio population, where 95% of people were found to be exposed to pesticides, with significant differences related to sociodemographic, lifestyle factors, and compound types [61].

The general population exposure to OCPs in different countries around the world was evaluated in many articles, but only Ntow et al. analyzed breast milk and blood samples obtained from Ghanaian farmers in which the levels of pesticides were the highest when compared to other studies [62]. 

Among the European population, OCP levels in blood samples of Italians, Spaniards, and Belgians were measured in some studies. In Italy, people living in a rural and an urbanized area were recruited. In their blood samples, 16 different OCPs were detected, with a predominance of p,p′-DDE and HCB that was strongly correlated with age, but not with gender [21]. These data were confirmed by the first work of Charlier et al., who measured them in the Belgian population in 2002 [63]. These researchers also evaluated OCP exposure in men with infertility problems, analyzing their semen and blood samples as well as blood samples from their mothers. There were no differences in OCP concentrations between fertile and unfertile men in either sample type and no correlation between semen quality and p,p′-DDE concentration. However, p,p′-DDE was more abundant in the mothers of infertile men than in those of normofertile ones, suggesting that male infertility could be associated with the mother’s exposure to p,p′-DDE during pregnancy, with deleterious effects restricted to intra-uterine life [64]. Also in Spain, in a study based on postmenopausal women undergoing different types of surgery, the levels of OCPs were similar to those in the articles mentioned above [65]. 

Among the Asian population, the Japanese, Chinese, Korean, and Lebanese populations were also investigated. In Japan, in the first years of the 21st century, 21 different pesticide chemicals were detected in pregnant women, including three chemicals with no history of use in Japan. In general, the bodily burden of OCPs is associated with age, pre-pregnancy body weight, and history of past conceptions [66]. Promisingly, pesticide levels dropped from levels found in previous studies in the Japanese city in question. 

Studies in China focused on women, in particular on young mothers. In 2004, Qu et al. monitored blood, breast milk from 90 young mothers, and cord blood from their sons. Levels of hexachlorocyclohexane (HCH) residues in human milk and maternal blood were relatively lower than those reported in other countries, whereas the levels of DDTs were much higher than those in Japan and most European countries. In addition, they were significantly lower than those in developing countries (Romania, Slovakia, Thailand, and especially India). In any case, pesticide residue levels decreased over time, suggesting that these compounds had come from the past usage [67]. In a very recent paper by Kuang et al., OCP concentration was monitored in young mothers’ breast milk in Jinhua, an inland and medium-sized city in China; OCP levels were generally found to be lower than those found in other towns or countries. The most detected compound was p,p′-DDE, followed by β-HCH and hexachlorobenzene (HCB), with a strong correlation with age [68]. In blood samples of a Korean sample population, the most detected compound was HCB, but the highest concentration was p,p′-DDE, with maximum peaks in men [69]. All these results were confirmed in a Lebanese sample population in which an inverse association between HCB concentrations and BMI as well as HCB, β-HCH, and DDE levels and smoking habits were found [70]. 

In Bolivia, high levels of exposure to p,p′-DDE were observed and serum p,p′-DDE concentrations were associated with residence time in the study area, personal hygiene after the working day, and BMI [71].

In an old paper by Selden et al., HCB levels in smelter workers were quadrupled compared to those in the control group and increased in relation to the years of exposure [72]. 

Another pesticide class is pyrethroids. In this case, pyrethroid metabolites can be monitored in urine; cis and trans-3-(2,2-dichlorovinyl)-2,2-dimethyl-(1-cyclopropane) carboxylic acid (DCCA), 3-(2,2-dibromovinyl)-2,2-dimethyl-(1-cyclopropane) carboxylic acid (DBCA), 3-phenoxybenzoic acid (3PBA), 4-fluoro-3-phenoxybenzoic acid (FPBA), and chrysanthemumdicarboxylic acid (CDCA) were detected in urine samples. There were three papers about pyrethroids, all focused on children. DCCA, 3PBA, and FPBA were the most frequently detected in urine samples in high concentrations in populations in the US and China. Primary routes of exposure to these compounds were found to be both dietary direct and indirect ingestion [73,74,75]. 

Glyphosate (GLY) is another widely used pesticide. It can be detected in urine with its metabolite aminomethylphosphonic acid (AMPA). The detection rates of GLY and AMPA in the urine samples of exposed workers was over 80% and the concentration of these compounds was positively correlated with the concentration of GLY in workplace air [76]. Instead, in the general population, GLY and AMPA were detected respectively as 31.8% and 40.1% in German young people [77] and as 27% and 50% in Slovenian children [76]. In both groups, urinary GLY and AMPA levels were higher in males than in females and they were correlated with age, because of their ability to bioaccumulate. 

The last POP category is perfluorinated alkylated substances (PFASs), which were very frequently detected in the serum of Czechs, although at levels relatively lower than those reported in other studies worldwide. The most occurring compound was perfluorooctanesulfonate (PFOS) [28]. 

The recurring result in all the papers analyzed in this review was the correlation between concentration of POPs in biofluids and the individual’s age, and this constitutes evidence of their persistence characteristic (as the name suggests) that determines their bioaccumulation. Another result was the reduction compared to the past; this is promising, but it is still not enough to prevent health consequences.

### 4.2. VOCs: Volatile Organic Compounds

Volatile organic compounds (VOCs) are compounds that have a high vapor pressure and low water solubility. Many VOCs are ubiquitous contaminants released into the environment by natural (i.e., they are released by plants, animals, and inorganic material) and human-made sources, including tobacco smoke, petroleum products, chlorinated water, and synthetic products such as paints, lubricants, and insecticides. VOCs include a variety of chemicals, some of which may have short- and long-term adverse health effects. The principal difference in POPs is that VOCs are not persistent and in fact they have a relatively short biological half-life (4 h) and are rapidly eliminated from the body [78]. However, this aspect does not make them less dangerous for human health: Repetitive or ongoing exposure can lead to an increase of endogenous VOC levels in the body. Long-term exposure to VOCs may increase risk for some types of cancers and birth defects [79]. 

Exposure to VOCs occurs mainly through inhalation, but also through ingestion and dermal contact. Human exposure occurs indoors and outdoors, but the concentration of VOCs in indoor environments is 10 times higher compared to outdoor environments. 

VOC biomonitoring is important for assessment of exposure to harmful VOCs, and it can be carried out using all biological fluids: exhaled air, blood, urine, human semen, saliva, breast milk, and so on. Since VOCs are volatile compounds, the most appropriate analytical extraction techniques are HS-SPME and thermal desorption. 

In selected papers focused on VOC analysis, mainly untargeted analysis on all detected volatile compounds was carried out by the authors. 

In five of six papers about occupational exposure, worker exhaled air was analyzed. 

VOC levels in the exhaled air of firefighters seemed to be strongly influenced by fire exposure; in fact, benzene, toluene, and styrene concentrations in exhaled air increased by at least two times post-exposure in both fire attack, victim search, and outside ventilation firefighting positions [80,81]. Workers from different kinds of industries were subjected to biomonitoring in relation to their exposure to VOCs. Jalali et al. found 40 different VOCs in men exposed to crystalline silica dust, in whose exhaled air the concentrations of acetaldehyde, hexanal, nonanal, decane, pentadecane, 2-propanol, and 3-hydroxy-2-butanone were higher than in healthy controls of smokers and nonsmokers [82]. In the workers in industries that use aromatic and chlorinated VOCs, the difference between exposed subjects and controls was very significant. Several VOCs were detected and quantified in all pre-shift urine samples, except for styrene, chloroform, dichloromethane, and toluene, which were present only in post-shift samples. Moreover, all volatile compounds were in higher concentration in post-shift urine samples than in pre-shift ones [83]. 

Even in less heavy jobs there could have been a very high exposure to dangerous VOCs. This was the case of nail technicians, who are exposed to a wide variety of chemicals during their work shift while using products to do manicures, pedicures, nail art, and artificial nails [84]. In fact, in 10 nail technicians, toluene and ethyl acetate levels in the blood were found to be higher in post-shift than pre-shift. On the other hand, methacrylates, which were detected in saloon air, were not measured in blood and exhaled air because of their instability. However, this does not exclude the presence of those residues. 

Another heavily exposed category was that of operating room personnel. In an untargeted analysis, 18 VOCs were identified in 13 staff units in which the concentrations of sevoflurane, dimethyl sulphide, and methyl methacrylate in exhaled air after surgical interventions were significantly higher than those found before surgery [85].

A more targeted analysis was carried out by different research groups that monitored the levels of the most used anesthetics, such as sevoflurane (SEV), isoflurane (ISO), halothane, isopropyl alcohol (IPA), and N_2_O in operating room and general hospital staff. In all these analyses, regardless of the absolute value of concentration, post-shift concentrations of these compounds were higher than pre-shift ones both in urine samples [86,87] and in exhaled air [88] and correlated with their levels in the operating room air. 

VOC biomonitoring in the general population has been widely discussed. Blood, urine, and exhaled air were the most used biological fluids, and our research group has the merit of having analyzed the VOCs of male semen for the first time. 

In 1994, in the US population 1,1,1-trichloroethane, 1,4-dichlorobenzene, 2-butanone, acetone, benzene, chloroform, ethylbenzene, m-, p-xylene, styrene, tetrachloroethene, and toluene were detected in most blood samples of non-occupationally exposed people [89], and later Jia et al. evaluated the median blood/air distribution coefficients of several VOCs detected in blood samples including methyl tert-butyl ether that remained unchanged even after smoking [90]. In the US population of Louisiana, VOC levels in two subpopulations were also compared. Thirty VOCs were detected in blood samples with a descending frequency for benzene and m-, p-xylene, 1,4-dichlorbenzene and toluene, ethylbenzene, and styrene [79].

Urine samples were used in three papers. In the first work, the authors screened VOC composition in 100 volunteers who participated in the cleanup work of the Herbei Spirit oil spill following an accidental oil spill that occurred near the Port of Daesan on the Yellow Sea Coast of Taean County in the west of the Republic of Korea on 7 December 2007 [91]. Some VOC metabolites were found at higher levels in the urine samples of volunteers after cleanup work; in particular, mandelic acid and trans,trans-muconic acid were present in even higher concentration in non-smokers. In general, the exposure effects were found to be stronger in women. 

In the second work, O’Lenick et al. detected 28 polar VOCs in urine samples from young men and women, with a predominance of several ketones (2-pentanone, 4-heptanone, 2-butanone, and 4-methyl-2-pentanone). Metabolites of phthalates were also found [92].

In the last work, toluene, ethylbenzene, xylene isomers, styrene, and p-dichlorobenzene were detected in morning urine samples with a strong correlation with time-weighted average air concentrations in the bedroom [93].

Only Delfino et al. evaluated VOC composition in exhaled air samples, in this case from children with asthma. In the exhaled air samples of asthmatic children, benzene, methylene chloride, styrene, tetrachloroethylene, toluene, m-, p- and o-xylene, and p-dichlorobenzene were detected in more than 75% of samples, with a positive association between benzene level and bothersome or more severe asthma symptoms [94]. 

Our research group was the first to perform VOC analysis on human semen. We analyzed volatile compounds in human semen from men with infertility problems; in these subjects we detected many VOCs with a high inter-individual variability. Nevertheless, we observed a correlation between some compounds and the motility of sperm cells; 2-methyl-1-pentene and propylyclohexane were exclusive to subjects with high motility sperm, whereas 3-methylbutyl acetate and 1-ethoxyethene were specific to subjects with slight asthenozoospermia, and o-pentylhydroxylamine and 2,6-dimethylpyrazine for severe asthenozoospermic samples [95,96]. 

Moreover, in young men we compared VOC fingerprinting of human semen, blood, and urine from the same subjects, demonstrating that human semen was richer in volatiles than the other biological samples and that the different bio-fluids can be classified based on their VOC composition [97]. 

Volatile halogenated hydrocarbons were detected in the late 1980s by Barkley et al. in exhaled air (n = 18), urine, and blood (n = 9) samples [98]. 

Traces of dichlorobenzidine (DCB) ranging from 1.6 to 8.9 ppb were detected in the same percentage in samples from DCB-exposed and non-exposed workers in a chemical plant [99], and the difference in p-octachlorostyrene (P-OCS) between exposed subjects compared to controls was far greater, with a good correlation between P-OCS level and the cumulative number of years of exposure to hexachloroethane [72]. 

There is extensive literature on BTEX (benzene–toluene–ethylbenzene–xylene). BTEX are mono aromatic VOCs and one of the most widespread chemicals produced globally. They are classified as hazardous air pollutants due to their potential to damage human health, so much so that the International Agency for Research on Cancer (IARC) has included benzene and ethylbenzene in the list of human carcinogens and possible carcinogens, respectively. Human health effects of exposure to BTEX are mainly dependent on exposure duration and concentration [100]. Rafiee et al. studied occupational exposure to BTEX in workers at composting facilities and in waste autoclave operators. In the first group, mean levels of urinary benzene, toluene, ethylbenzene, and m-, p-, and o-xylene in the exposed subjects were 1.4 to 3.7 times higher than the values in the control group. Furthermore, post-shift levels were significantly higher than pre-shift levels for all chemicals, despite the use of personal protective equipment [100]. In the second group, the exposed workers had significantly higher urinary BTEX levels (2.5-fold) than the controls, except for ethylbenzene. There was a significant relationship between the amount of generated waste per day and the urinary BTEX [101].

For the general population regarding exposure to BTEX, there were two old publications. In 1999, the exposure of a group of Italian cyclists to BTEX was evaluated comparing pre-run and post-run BTEX levels. After a 2 h bike run in a polluted urban city, benzene and toluene had significantly increased in blood, and toluene and xylenes in urine [102]. Urban traffic also influenced benzene level in urine samples from urban traffic policemen, who had end-of-shift benzene concentrations much higher than pre-shift concentrations [103]. 

In 1989, in a German study on smokers and non-smokers, benzene and toluene were higher in smokers and BTEX were detected in all blood samples, reflecting the ubiquitous exposure of humans to these agents in an urban environment [104]. 

In a recent study, specific biomarkers for toluene, ethylbenzene, m- and p-xylene (o-cresol, 2-ethylphenol, methylbenzylalcohol, and 4-methylbenzylalcohol) were detectable in all urine samples from children between 4 and 15 years old [105]. 

Because of its carcinogenicity, exposure to benzene was shown to have deepened in some papers. In workers exposed to different levels of benzene, a linear correlation between air concentration and exhaled air was found [106]. It was also noticed that despite benzene levels in smokers’ exhaled air, samples were higher than those of non-smokers, and in non-smokers ambient air benzene concentration was lower than in non-smokers’ exhaled air samples, suggesting an additional source of benzene from outdoor ambient air derived from smoking itself [107]. 

Even in glue- and shoe-making factories, there is a very high exposure to benzene. In fact, levels of benzene after work shifts were significantly higher than before the shift, and both were higher than in unexposed subjects [108]. 

Other interesting VOCs are acrolein, methyl tert-butyl ether (MTBE), cyclophosphamide, and n-alkanes. A strong correlation was observed between the concentration of acrolein in indoor air and exhaled air, leading to acrolein in exhaled air being suggested as an environmental exposure biomarker [109]. MTBE, together with benzene, was present in urban traffic policemen’s urine in very high concentrations [98]. Fortunately, n-alkanes derived from fuel emissions were detected above the detection limits only in 1.3% of 1200 blood samples from the American population [110]. Finally, cyclophosphamide was found in urine obtained from hospital staff [111].

#### Gas Sensors for VOC Detection 

The gas chromatography/mass spectrometry system remains the chosen analytical method for studying volatile composition in clinical and environmental analysis, but the development of chemoresistive gas sensor-based devices using sensor response patterns for VOCs could represent a real turning point in fast exposure assessment. Indeed, sensor technology allows for portable and low-cost devices for large-scale screening. As gas sensors have been exploited for VOC detection in applications involving environmental pollution monitoring in indoor and outdoor air analysis, as well as in biomedical applications for biospecimen analysis targeted to diagnostics, they are a consolidated and advanced technology that is ready to be applied in human biomonitoring. As the concept of human biomonitoring is relatively new for the sensor sector, at present only a few works based on gas sensors and gas sensor systems (i.e., e-noses) have been published regarding strictly HBM studies, whereas most of the literature regards the closer field of medical or clinical applications in relation to VOC analysis in biospecimens. Gas sensors can take up the challenge of human biomonitoring. The use of gas sensors as a tool complementary to GC/MS would lead to increased recognition of biomonitoring as the most informative technique, and greater dissemination of HBM programs in all nations. 

The use of gas sensors would lead to an increased recognition of biomonitoring as the most informative technique, and a greater spread of HBM programs in all nations.

The interest in VOC investigation has grown over the past 20 years.

Many VOCs are categorized by the WHO as indoor pollutants; some are components of products commonly used indoors, whereas others result from chemical reactions or are reactive precursors of secondary products [112]. Many VOCs cannot be attributed to a particular source. The totality of VOCs entering the human body contributes to the internal dose of exposure to these xenobiotics. 

In this review, we presented the works that were filtered by the PRISMA protocol set for our meta-analysis, although many other papers concerning gas sensors and e-noses analyzing VOCs of human specimens for related diagnostic applications were excluded.

Publications regarding related diagnostic medical applications were excluded from the review.

In a recent study, the research group of Jaeschke designed an e-nose-based analyzer composed of three arrays of commercial metal oxide (MOX) gas sensors based on both analog and digital technology. This device is able to detect acetaldehyde, acetone, ethanol, ethyl acetate, isoprene, and n-pentane in a range between 3 and 18 ppm without the interference of humidity, which is important for its application to biological fluids that are mainly composed of water [113]. MOX-based sensors were also used in an e-nose of Pace’s research group for the detection of acetone, ammonia, carbon monoxide, and carbon dioxide, with other types of sensors: an electrolyte sensor, a temperature sensor, and a humidity sensor [114]. Jayasree et al. applied an array of three MOX sensors based to a tin dioxide semiconductor for the detection of acetone in the exhaled air of patients with diabetes mellitus [10]. Ethanol and acetone, together with methanol and ammonia, were also detected in human exhaled air by an optical electronic nose system [115].

In a work from 2017, Liu et al. developed a smartphone-based sensing system for real-time acetone, ethanol, acetic acid, and formaldehyde monitoring in exhaled air using alternative current impedance measurement using interdigital electrodes modified with graphene, ZnO, and nitrocellulose membrane [116]. In the same year, Shao et al. developed a sensor device with PtOx/GQDs/TiO_2_ nanoporous thin film to detect benzene, toluene, and isopropanol in exhaled air [117]. 

Hoppe et al. presented their results of the detection of 100 ppm of ethanol, 2-propanol, and n-butanol obtained by chemical sensors; the sensors based on (CuO-Cu_2_O)/ZnO:Al film showed a reduced dependence of the sensing properties on relative humidity, which is very important for both ambient gas-sensing applications and VOCs detection in human exhaled air [118]. 

A surface acoustic wave (SAW) transducer device was designed in 2021 by Kus et al. using gold nanorods and silver nanocubes functionalized with thiol and modified with Calix[4]arene, which increases the sensor responses by six- to eight-fold, with a selectivity towards chloroform and toluene, respectively. Detection levels reached 80–100 ppm [119].

Gas sensors that respond to toluene have also been extensively studied. In addition to the mentioned works [118,119], toluene was detected in human exhaled air by porous films made of Au-loaded TiO_2_ nanotubes [120], by Pd-SnO_2_-clustered nanoparticles [121], and by a synergetic p + n field effect transistor (FET) amplification circuit [122]. The latter system was more specific for xylene and also showed good selectivity for formaldehyde [122]. 

Quartz tuning fork (QTF)-based sensors were also used for the detection of 1,4-dimethoxy-2,3- butanediol, cyclohexanone, acetone, ethanol, and methanol in exhaled air [123].

All these papers investigated VOCs in exhaled air; on the other hand, Berkhout et al. analyzed fecal VOCs in infants, aiming to evaluate the sampling conditions and environmental factors influenced by eNose Cyranose320@, composed of an array of nanocomposite sensors and advanced pattern recognition algorithms [124]. 

In only two of our papers, already discussed in previous section, both GC/MS and gas sensors were used on the same samples. In particular, we analyzed human semen VOCs with a MOX gas sensor inserted at the end of a GC capillary column by a splitter. Using generated sensorgrams (i.e., dynamic sensor signals acquired during VOC elution from gas chromatographic column), we were able to discriminate samples from patients with different spermatic mobility with an accuracy of 77–78%. Furthermore, comparing the sensorgrams to chromatogram signals vs. time, we noticed that the sensor showed different affinities to organic compound classes, with a higher reactivity with aldehydes, ketones, and acetamides [95]. 

In a second paper of ours, we used an array of four MOX sensors to detect VOCs in blood, urine, hair, and human semen samples from young men living in the “Land of Fires,” a contaminated area in southern Italy. Significant results related to human contamination were obtained from blood, urine, and semen analysis, yet the hair was less informative due to its dry matrix characteristic. 

Although we had a lower volume of human semen, the gas sensor responses to the VOCs in human semen samples were found to be elevated, confirming the GC/MS results that detected a VOC composition in human semen that was much more abundant than other biological fluids [97].

### 4.3. Other Pollutants

There are other air pollutants that are not attributable to POPs and VOCs. 

Aromatic toluene diisocyanate (TDI), the monomeric methylenediphenyl diisocyanate (MDI), and polymeric MD are the most common diisocyanates to which workers are exposed via the thermal degradation of polyurethane products. Urinalysis of diisocyanate-derived amines can be used to quantify the often-intermittent exposure to isocyanates, which were quantified in all urine samples of exposed workers and which conversely were very low in non-exposed categories [125].

Phthalates and, in particular, bisphenol-A (BPA), are known for their capacity to be endocrine disruptors, and for this reason their use has been dramatically dropped. BPA was found in the urine and blood of workers at a hazardous waste incinerator [126], but also in the blood of non-occupationally exposed women, with a predominance in infertile women compared to fertile ones (77% vs. 29%, respectively) [127]. 

Hexahydrophtalic anhydride (HHPA) urinary levels in Swedish exposed workers were higher in post-shift than in morning urine samples and were higher than those in the urine samples of non-exposed men [128]. Moreover, N-methyl-2-pyrrolidone (NMP) and its metabolite (5-hydroxy-N-methyl-2-pyrrolidone) were more abundant in post-shift than in pre-shift urine derived from workers of glue and adhesive compound production plants [129]. 

Parabens are widely used as preservatives in personal care products, foodstuffs, and pharmaceuticals, but they have a potential endocrine disruption effect. HBM was used to estimate the cumulative exposure of humans to these pollutants using urine samples. Methylparaben, ethylparaben, propylparaben, and butylparaben were detected in all analyzed samples. They were significantly higher in women than in men and they were correlated with BMI and age [130]. 

### 4.4. Biological Matrices and Biomarker Extraction Methods in HBM

**Figure 4:**
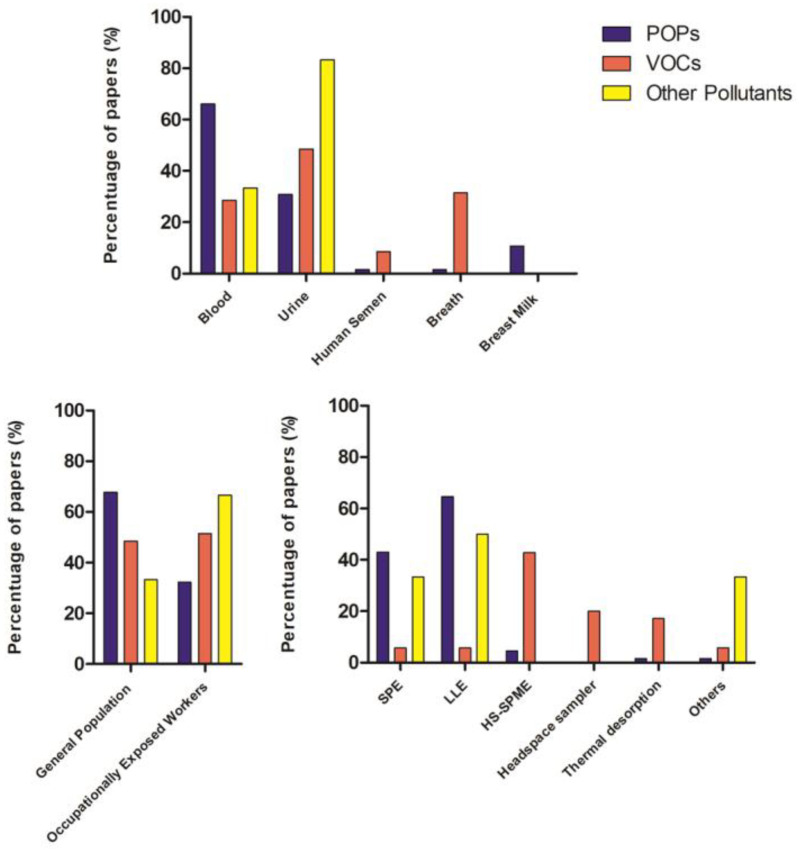
Column charts related to biological fluid, type of population, and extraction method reported in the papers of the systematic review in relation to pollutant classes.

As can be seen from the collection of works, different biological matrices were used (Figure 4).

In HBM, the selection of the preferred matrix depends on the physical–chemical properties of the chemical compound that influences its metabolism and excretion routes. Blood and urine are most likely the most used and widely accessible biofluids, but other matrices have also begun to be used. The choice of extraction and detection method also depends on the type of chemical substance whose internal dose is to be determined. 

In general, blood is the preferred matrix for many chemicals, as it is in continuous contact with the whole organism and is in equilibrium with organs and tissues; it is used for the determination of POPs [14,15,16,17,18,19,20,21,22,23,24,25,26,27,28,29,30,31,32,33,34,35,38,39,41,42,54,55,56,58,59,62,63,64,65,66,67,69,70,71,72] with the exception of some POPs [39,40,44], PAHs [45,46,47,48,49,50,51,53], and some pesticides (i.e., pyrethroids) [60,61,73,74,75,76,77,78] that accumulate more in urine. Blood analysis has a standardized sampling procedure and it is generally the most widespread analysis in HBM studies, but it has some limitations, such as the invasiveness of sampling and the need for the presence of professional operators. In general and specific populations, exhaled air and breast milk were also studied. To extract these chemical pollutants from biological matrices (blood, urine, breast milk) liquid–liquid extraction (LLE) or solid phase extraction (SPE) can be used. In some cases, a combination of the two methods was applied. Urine, breast milk, and exhaled air sampling are non-invasive, easy, and can be done directly by the individuals. Urine is the most useful matrix for measuring urinary biomarkers that are rapidly metabolized and excreted, such as non-persistent pesticides, bisphenol A (BPA) [126], and other phenols [44], parabens [130], volatile organic compounds (VOCs) [83,85,87,92,93,97,98,99,100,101,102,103,105,108,111], and PAHs [45,46,47,48,49,50,51,53,55,60,61,73,74,75,76,77,78]. However, urine analysis suffers from great temporal variability in composition within individuals.

Due to its volatile nature, exhaled air is preferred to analyze VOCs, followed in the last few years by urine. VOC extraction is based on absorption/desorption phenomena on a specific substrate coated with an extractive phase that captures the compounds and then releases them at high temperatures. Thermal desorption and HS-SPME is the most used extraction method for VOCs.

## 5. Conclusions

HBM allows for the measurement of personal total exposure to a specific chemical by measuring the un-metabolized and/or metabolized compounds in biological fluids. HBM has always played an important role in assessing and managing chemical risks of environmental exposure both in occupationally exposed workers and in the general population. Over the years, HBM has established itself and spread throughout various nations as a tool for environmental policies thanks to the use of increasingly advanced technologies that give HBM the ability to provide fundamental information on human exposure to environmental pollutants. All available biological fluids are useful to determine the bodily burden of all types of pollutants, starting from the most used blood and urine and moving to human semen, exhaled air, saliva, breast milk, and so on. 

GC/MS represents one of the most valid tools for human biomonitoring, combining an excellent separation method with a good detection system. Depending on the type of compounds, it is important to add an appropriate extraction method to have a complete chemical analytical methodology. The current review of the literature selected by the defined PRISMA protocol allowed us to survey the main findings of HBM studies. From this systematic review, a worrying scenario of human contamination emerged that includes not only the occupationally exposed population but also the general population. The general population living in industrial/agricultural contaminated areas showed levels of contaminants comparable to occupationally exposed population living in the same areas, and higher than those of the general population living in other, non-contaminated areas. No one is excluded from the harmful effects of pollution, including fetuses and newborns; exposure to environmental hazardous chemicals occurs through life with related adverse effects accumulating over time. The most alarming result was related to the entire class of POPs that, as documented by the publications in this review, accumulate along the lifetime with a clear association with individual age. The analysis of VOCs in human biofluids is a wide, not-fully-explored field. HBM studies offer the opportunity to found novel VOC markers of environmental pollution. In our review, the contamination of human biofluids by VOCs revealed a worrying situation, considering that a lot of VOCs are used in a wide range of household and industrial products. They are ubiquitous, and human exposure to VOCs is indeed pervasive. Although they have a relatively short biological half-life, some of them can have toxic effects and can elicit adverse effects in the population. 

Moreover, for many of the chemicals belonging to all three groups of pollutants, in the publications that measured them in occupationally exposed workers, post-shift levels were significantly higher than pre-shift; this is evidence of the higher health risk for special categories of workers. 

Finally, what emerged was a particular concern for the most vulnerable population groups (pregnant women, fetuses, and children). 

Gas sensors/gas sensor arrays are a complementary technology that can integrate GC/MS in HBM studies. In the future, once trained with GC/MS data, they can predict the level of specific VOCs in different biological matrices used in HBM, and hence become intelligent devices for the qualitative and quantitative analysis of complex VOC mixtures. It would be strategic to develop and use these new technologies to allow for faster and cheaper analyses that would ensure early and large-scale biomonitoring. 

It is now widely recognized by the global community that monitoring the chemical bodily burden of citizens and assessing the associated health impacts has to become the new approach to prevent adverse effects of pollution on public health. However, to achieve such an ambitious goal, a sustainable surveillance system is needed as well as a system embedded in legislation that can be used to measure the internal dose of harmful chemicals via human biomonitoring. More standardized procedures and guidelines as well as coordination between the different international institutions are needed. The multiplication of HBM programs in political initiatives is the necessary parameter that will determine the success of HBM. Low investment by politicians in HBM tools will obviously limit the new knowledge that can be acquired.

Finally, in the near future it is desirable for HBM to be used more and more in epidemiological studies in combination with health data to demonstrate an association between the bodily burden of pollutants and their health effects. Furthermore, the integration of such studies into adverse outcome pathway science will take us one step closer to understanding the causal links between exposure and health effects.

## Data Availability

Not applicable.

## Supplementary Materials

The following are available online at https://www.mdpi.com/article/10.3390/ijerph181910236/s1, Table S1: Summary of included studies concerning Persistent Organic Pollutants (POPs), Table S2: Summary of included studies concerning Volatile Organic Compounds (VOCs), Table S3: Summary of included studies concerning pollutants different from POPs and VOCs, Table S4: Summary of included studies in which gas sensors are used for VOC detection.
