# Supplementary material for: Human Biomonitoring of Environmental and Occupational Exposures by GC-MS and Gas Sensor Systems: A Systematic Review
Source: Int J Environ Res Public Health. 2021 Sep 29;18(19):10236. doi: 10.3390/ijerph181910236 (PMC8508139; doi:10.3390/ijerph181910236)
Supplement: Supplementary file 1 [file ijerph-18-10236-s001.zip › ijerph-1273989-supplementary.pdf]

International Journal of
Environmental Research and Public Health

MDPI

Table S1. Summary of included studies concerning Persistent Organic Pollutants (POPs).

Pollutant Abbreviation	[ref.] First Author (Years)	Study Design	Biological Fluid	Country (Region or City) and Sampling period	Population and Group Characteristics [N, Type, n, (sex, age)]	Inclusion (IC)/ Exclusion (EC) Criteria	Extraction Methods (Solvent and Ratio) and Limit of Detection (LOD) and/or Quantification (LOQ) (ng/L)	Main Findings
	**General Population**							
POPs	[14] Luzardo (2019)	CSS	Blood (serum)	Romania (Brasov)<br>December 2017 February 2018	**N=121**(29M/92F, 27.8 ± 9.8 y)	NA	SPE<br>LOQ: 100.00<br>PCBs, p,p′-DDE: 50.00	• Older subjects showed highest serum levels of OCPs and PCBs;<br>• Lower serum levels of p,p′-DDE, HCH and PCBs in Romania compared to the past;<br>• Phenanthrene and naphthalene suggest an unknown source of exposure
	[15] Rudge (2012)	CSS	Blood	Brazil (São Paulo) 2007-2008	**N=155** Pregnant women (F, 26 ± 5.4 y)<br>n=36 Rural 1 (F, 29.1 ± 6.4 y)<br>n=19 Rural 2 (F, 24.6 ± 6.8 y)<br>n=20 Urban 1 (29.4 ± 7.6 y)<br>n=20 Urban 2(F, 22.9 ± 4.5 y)<br>n=20 Urban 3 (F, 25.4 ± 6.3 y)<br>n=20 Industrial (F, 24 ± 6.5 y)<br>n=20 Coastal (F, 26.1 ± 6.2 y)	**IC:**<br>• women who were admitted to the delivery rooms at seven maternity hospitals<br>• living in the specific study site for at least 1 year prior to the pregnancy but duration	LLE<br>(n-hexane)<br>LOD: 0.40 -12.00<br>p,p-DDT, PCB156: 41.00	• PCB levels in Brazilian delivering women were higher than in the plasma of delivering women in South Africa and Vietnam but lower than in Canada, the Netherland and Spain;<br>• p,p′-DDE metabolite was detected in 100% of maternal blood samples and p,p′-DDT metabolite in only 20% of the samples;

						at the present address was also recorded		• Higher levels of pesticides in blood of delivering women living in rural areas; • The body burden of selected POPs increases with age
	[16] O'Brein (2012)	CSS	Blood (plasma)	Canada (Nunavik) 2006-2008	**N=155** Children (79M/76F, 25.2 ±9. 6months)	NA	SPE followed by LLE (dichloromethane) LOD: 5-300	• PCB-153 was detected in all participants, whereas PCB-118, PCB-180, p,p′-DDE, cis-nonachlor, and oxychlordane were detected in at least 70% of samples; • Some emerging POPs, especially BDE-47, -99, and -100, were detected in a larger proportion of our participants • PBDE concentrations is higher in our children compared to Nunavik adults • PFASs, PFOS was detected in all participants of the present study
	[18] Jogsten (2010)	CSS	Blood	Sweden (Örebro) 2007	**N=9** (5M/5F, 19-65y)	NA	Weak anion exchange SPE LOD: $4x10^4$	• Highest concentration of both PBDFs and PCDD/Fs were obtained from a 61 year old female • PBDE levels vary with no signs of age or gender dependence
**Phenolic organohalogens**								
	**General Population**							
POHs	[19] Dufour (2016)	CSS	Blood (serum)	Belgium	**N=20** (14M/6F, 21 – 69 y)	NA	SPE followed by LLE(hexane) LOD: 0.67 – 1.51 2,4,6-TBP: 15.00 PCP: 13.50	• The method developed allows the simultaneous determination of PCP, TBBPA, bromophenols, OH-PBDEs and OH-PCBs in human serum samples;

								• TBBPA, bromophenols or OH-PBDEs were not detected in any analysed samples;<br>• The predominant POH found in the serum was PCP;<br>• OH-PCBs were detected in all samples except 4-OH-CB 153 and 3-OH-CB 180 which were found in 80 and 65% of the samples respectively
**Polychlorinated biphenyl (PBCs)**								
	**General Population**							
PCBs	[20] Humblet (2010)	CSS	Blood (serum)	Russia 2003 - 2005	**N=446** (F, 23–52 y)	NA	SPE	• PCB concentrations increased significantly with age, residential proximity to a local chemical plant, duration of local farming, and consumption of local beef;<br>• PCB concentrations decreased with longer breastfeeding, recent increases in body mass index, and later blood draw date.
	[21] Turci (2009)	MCS CSS	Blood (serum)	Italy (Pavia and Novafeltria)	**N=95**<br>n=59 from Pavia (36M/23F; mean age 41.7 y)<br>n=36 from Novafeltria (19M/17F; mean age 42.3 y)	**EC:** History of occupational or accidental exposure to PCBs and OCPs	LLE (ethyl ether/hexane 1:1, v/v)<br>LOD/LOQ: 50.00- 150.00	• A cost- and time-saving screening procedure for the determination of 15 PCB in human serum was developed and validated;<br>• A correlation between age and concentrations of the more persistent PCB, p,p’-DDE, and HCB exists;<br>• Since no statistical difference was observed between males and females;

							• PCB concentrations between the two geographical areas may be related to different dietary or agricultural practices
[24] Parera (2013)	MCS CSS	Blood Breast milk	Spain (Mataró and Arenys de Mar) March 1995 - June 1995, 2005, 2008	**N=258** For blood<br>n=68 Exposed from Mataró (32M/36F, 48.5 ± 15 y)<br>n=94 Controls from Mataró (47M/47F, 48.3 ± 14 y)<br>n=86 from Arenys de Mar (50M/36F, 48.2 ± 13 y)<br>n=10 Workers (7M/3F, 43.9 ±12 y)<br>**N=54** for breast milk<br>n=15 ⩽30 years old, 1st child (26.2 ± 3.4 y)<br>n=11 ⩽30 years old, 2st child n=11 (27.72 ± 3.1 y)<br>n=15 >30 years old, 1st child (34.8 ± 2.9 y)<br>n=13 >30 years old, 2st child (35.0 ± 3.3 y)	**IC for Exposed group:** Living less than 1 km from the incinerator plant<br>**IC for Control group:** Living more than 3 km from the incineration plant	For blood: Chromatographic glass column<br><br>For breast milk: LLE (petroleum ether, diethyl ether)	• PCBs levels consistently decrease since 1999;<br>• No differences in blood levels of these compounds between subjects considered exposed and non-exposed to solid waste incineration plant were observed; main route of contamination of these compounds to humans is through other sources, mainly diet.
[22] Koppen (2001)	MCS CSS	Blood	Belgium (Peer and Antwerp) June 1999 - September 1999	**N=200** (F, 50-65)<br>n=100 from Antwerp<br>n=100 from Peer	**IC:**<br>• Non- or ex-smoker,<br>• Minimal living time of 10 years in the study area<br>• Working in the town of residence or at home.<br>**EC**: Jobs with specific risks of exposure.	Soxhlet apparatus with n-hexane:acetone (2:1, v/v)<br>LOD: 10.00-20.00<br>PCP: 70<br>(10 ng/g lipids)	• PCB congeners concentrations are higher in the women living in the urban area than in rural one.

[23] Inoue (2006)	MCS CSS	Blood Breast milk	Japan (Hokkaido, Miyagi,Gifu and Hyogo)	**N=89** (F, 30.1 ± 4.6)<br>n=20 from Hokkaido (F, 27.7 ± 4.8)<br>n=40 from Miyagi (F, 30.7 ± 4.1)<br>n=20 from Gifu (F, 30.0 ± 4.3)<br>n=9 from Hyogo (F, 33.3 ± 4.5)	NA	LLE (ammonium sulfate, ethanol, hexane 3:1:2, v/v)<br>LOD: 1 pg/g lipids	• Geographic location within Japan and the duration of nursing were discernible determinants for PCB levels in human serum and milk.
[17] Schettgen (2011)	CSS	Blood (plasma)	Germany May 2003 - January 2004	**N=105** (51M/54F, 5 -84 y)	NA	LLE (n-hexane) followed by clean up<br>LOD: 10.00	• The method developed allows to determine background burden of the general population to dioxin-like PCBs;<br>• Values obtained for the indicator PCBs 138, 153 and 180 (and HCB as well as p,p'-DDE) in the plasma samples are well below the reference values evaluated for plasma by the German Environmental Agency;<br>• HCB, DDE and PCB 138, 153 and 180 in the age group of 20–30 are in good congruence to the median values of other studies;<br>• The children aged less than 10 years showed a slightly higher internal exposure to PCBs compared to the persons aged between 11 and 20 years
[26] Charlier (2004)	CCS	Blood (plasma)	Belgium	**N=120**<br>n=60 Breast cancer women<br>n=60 Controls	**IC for Breast cancer group:**<br>• Diagnosis of breast cancer<br>• Surgical intervention	LLE (acetonitrile, potassium carbonate, petroleum ether/diethyl ether 98:2, v/v) and clean up	• PCB153 and PCB138 were found in significantly higher concentration in blood samples of women diagnosed with breast cancer when compared to a control group;

						**IC for Control women:** Healthy women		• PCB153 is a significant risk factor of breast cancer.
	**General Population**							
NDL-PCBs	[27] Kobayashi (2017)	CSS	Blood	Japan (Sapporo) 2002 - 2005	**N=169** (77M/92F) n=77 Mothers of male infants(mean age 30.5 ± 5.0) n= 92 Mothers of female infants (mean age 30.1 ± 4.)	**EC:** Miscarriage/stillbirth, relocation, voluntary withdrawal, and multiple births	NA	• Presence of NDL-PCBs in maternal blood during pregnancy has a gender-specific effect in increasing H19 and LINE-1 methylation levels in cord blood, particularly in female infants; • These associations were only significant among all infants of primiparous mothers.
**Flame Retardants (FRs)**								
	**General Population**							
BFRs	[28] Sochorová (2017)	MCS CSS	Blood (serum)	Czech Republic (Prague, Ostrava, Liberec and ˇZd'árnad Sázavou) 2015	**N=300** (171M/129F, 18 -65 y)	**IC:** Living in the studied area for at least 3 years	SPE LOQ: 0.004 - 0.364	• BFRs were quantifiable in only 9% of analysed serum samples; • The highest concentrations were observed for PBDEs, especially for BDE-47, BDE-99, BDE-153 and BDE-209.
	[29] Lenters (2013)	MCS CSS	Blood (serum)	Greenland, Poland and Ukraine August 2002 - February 2004	**N=299** Male partners of pregnant women n= 99 from Greenland (M, 32.0 ± 7.2 y) n=100 from Poland (M, 30.4 ± 3.8 y) n=100 from Ukraine (M, 27.6 ± 5.3 y)	**IC:** (a) Older than 18 years (b) Born in the country	Automated SPE LOQ: 0.02 to 0.12 ng/g lipids	• BFR body burdens were substantially higher in Greenlandic compared to European men, yet lower than concentrations reported for populations from the US; • The associations between ΣPBDEs and age were inconsistent.
	**Occupationally Exposed Workers**							

PBDEs								
	[30] Qu (2007)	CSS CCS	Blood (serum)	China (Guang-dong) NA	**N=55** n=20 Electronic waste dismantling workers (17M/3F, 18-45y) n=15 Exposed residents (12M/3F, 29–59 y) n=20 Controls (F, 28–35)	**IC for Exposed Worker group:** Working in waste dismantling **IC for Exposed resident group:** Reside in villages near the sea in South China, about 50 km from the electronic waste dismantling region **IC for Control group:** Perform work with no specific potential occupational exposure to PBDEs or any other chemicalexposure or other factors.	LLE (hexane/ methyl tert-butyl ether 1:1, v/v) LOD: 0.01 to 0.09 ng/g lipids BDE-209: 1 ng/g lipids	• Electronic waste dismantling workers had high concentrations of PBDEs, especially BDE-209; • Also BDE-197, BDE-207 and BDE-208 showed elevated concentrations in dismantling workers.
	[32] Jakobsson (2002)	CSS CCS	Blood (serum)	Sweden 1999	**N=70** n=19 Computer technicians (15M/4F, 27–45 y) n=51 Controls (F, 25-61 y)	**IC for Exposed group:** occupationally exposed to flame retardants in electronic equipment **IC for Control group**: No occupationally exposed to flame retardants	LLE (hexane/ methyl tert-butyl ether 1:1, v/v)	• Elevated serum levels of BDE-153, BDE-183 and BDE-209 were associated with computer work; • Estimated half-life of BDE-183 is 86 days and of BDE-209 6.8 days; • There was a clear correlation between BDE-153, BDE-183, and octa-BDE:2 and the duration of computer-work. • Contaminated food, particularly fish, is the main exposure route for PBDEs

[31] Sjodin (1999)	CSS	Blood (serum)	Sweden NA	**N=59** n=20 Computer clerks (20F, 25-61 y) n= 19 Electronics dismantlers (15M/4F, 25-60 y) n=20 controls: Hospital cleaners (20F, 30-60 y)	**IC:** Occupationally exposed to PBDE to derks working filltime at computer screen and persomnnel at an elecnics-disntling plant	LLE (hexane/ methyl tert-butyl ether 1:1, v/v) LOD: BDE-209: 0.3 pmol/g lipids BDE-209: 0.7 pmol/g lipids	• The major compound in personnel at the dismantling plant was 2,2',3,4,4t, 5', 6-heptaBDE and its concentration are almost twice as high as for 2,2',4,4'-tetraBDE in these workers and seventy times the level of this heptaBDE in deaners; • Decabromodiphenyl ether is bioavailable; • Occupational exposure to PBDEs occurs at the electronics-dismantding plant via inhalation of airborne particulate matter.
[33] Yu (2010)	CSS	Blood (serum)	China (Guiyu)	**N=6** Electronic waste dismantling workers	**IC:** Occupationally exposed to PBDEs	LLE (hexane/ methyl tert-butyl ether 1:1, v/v) by SPE LOD BDE-209: 40 pg	• Higher brominated diphenyl ethers could be oxidatively metabolized into OH-PBDEs and accumulate in human blood serum.
**General population**							
[23] Inoue (2006)	MCS CSS	Blood Breast milk	Japan (Hokkaido, Miyagi, Gifu and Hyogo)	**N=89** (F, 30.1 ± 4.6) n=20 from Hokkaido (F, 27.7 ± 4.8) n=40 from Miyagi (F, 30.7 ± 4.1) n=20 from Gifu (F, 30.0 ± 4.3) n=9 from Hyogo (F, 33.3 ± 4.5)	NA	LLE (ammonium sulfate, ethanol, hexane 3:1:2, v/v) LOD: 0.2-2 pg/g lipids	• BDE-209 was the PBDE detected at the highest concentration in serum of Japanese lactating women; • BDE-209 excretion in milk was lower than that of BDE-47 and BDE-153; • Geographic factor was the major determinant of PBDE levels in Japan; • The levels of PBDEs in Japan were much lower than those in the United States,

							Canada, and Mexico but similar to those in European countries.
[37] Guo (2020)	CSS	Cord blood	China (Sheyang) June 2009 - January 2010	**N=636** n=318 Mother (F, 285 <35 y, 33 >35y) n=318 Children (180M/138F)	**EC:** • Adverse pregnancy complications, • Twin birth	SPE LOD: 0.208 - 0.65 BDE-209: 10.03 LOD: 18.1 ng/g lipids	• BDE-209 was the most abundant congener of PBDEs; • Cord serum BDE-153 and BDE-154 concentrations were negatively associated with body mass index score and waist circumference at 7 years of age, with sex-specific effects; • BDE-154 concentrations in cord blood were related to a lower risk of childhood adiposity.
[34] Xu (2018)	CSS	Blood (serum)	China (Shanghai)	**N=25** (7M/18F, 40-75 y)	NA	SPE LOD: 0.010–0.030 ng/g lipids	• In human serum samples there are low concentrations of PBDEs compared with other cities and moderate levels of contamination with OH-PBDEs compared with other animals; • BDE-47 and 6-OH-BDE-47 were found to be the most prevalent congeners in these samples; • PBDE congener correlated with others suggesting that these compounds share similar sources and metabolic processing; • No significant correlations were found between the age, sex, or the workplace of the donors and concentrations of PBDEs/OHPBDEs.

[38] Hertz-Picciotto (2011)	CCS	Blood	USA (California) June 2003 - September 2005	**N=94** Autism children<br>n=49 Autism/autism spectrum (45M/4F, 3.7 ± 0.1)<br>n=24 Developmental delay (16M/8F, 3.7 ± 0.1)<br>n= Typical development 21(18M/3F, 3.7 ± 0.1)	**IC for all groups:**<br>• Children between two and five years of age,<br>• Reside in the study catchment area,<br>• Birth in California,<br>• Biological parent who speaks either English or Spanish.<br>**IC for Autism group:** Final diagnosis of autism by the Autism Diagnostic Observation Schedule and Autism Diagnostic Inventory-Revised<br>**IC for Developmental delay**: Diagnosis using the Mullen's Scales of Early Learning (MSEL) and the Vineland Adaptive Behavior Scales (VABS).<br>**IC for Typical development:** Children from the general population whose Composite scores on the	SPE<br>LOQ:<br>BDE-47: 0.03 ng<br>BDE-207: 0.015 ng<br>BDE-209: 0.01 ng<br>all other BDE: 0.006 ng.	• Levels of PBDEs may be sufficiently high that all children who are predisposed to develop autism have had exposures sufficient to surpass their individual thresholds.<br>• There are no association between children's circulating levels of PBDEs and autism case status

					MSEL and VABS were at or above 70.		
[35] Lunder (2010)	CSS	Blood	USA September 2006 -January 2007	**N=40** n=20 Mothers (F, 28.9 – 44.4 y) n=20 Children (8M/12F, 1.5 – 3.9 y)	**EC:** Not lived continuously in the United States since the child's birth.	LLE (hexane/ methyl tert-butyl ether 1:1, v/v) LOD: 0.0002-0.003 ng LOQ: 0.0021-0.0294 ng	• PBDEs for children were typically 2.8 times higher than for mothers, with median child:mother ratios varying from 2 to 4 for individual congeners; • BDE-209 was quantitated in 13 children and 9 mothers; • Children's increased hand-to-mouth activity, dietary preferences, and exposures from breast milk may result in greater ingestion of PBDEs than adults.
[36] Kim (2011)	CSS	Cord blood Breast milk	Korea (Seoul) 2008 - 2009	**N=111** n=90 Umbilical cord blood samples n= 21 Breast milk samples	NA	SPE LOQ: 0.5-1.0 ng/g lipids	• PBDE concentration were similar to Asian or European PBDE concentration and lower than US concentration; • The PBDE distribution pattern in both samples were quite similar to other foreign reports; • The most dominant congener was BDE47, while BDE100, 99, and 153 were also highly detected in both samples with slight differences; • A weak correlation between thyroid hormone and PBDE accumulation was observed in breast milk samples for BDE-153 and -28;

								• Cord blood samples displayed positive correlation with environmental surrounding factors (oil paint or insecticides), while breast milk samples displayed positive correlation with dietary intake (frequency of consuming fish or tea).
	[39] Ho (2015)	CSS	Urine Blood (plasma)	China (Hong Kong) March 2010 - July 2010	**N= 100** (50M/50F, 54.9 ± 21.9 y)	NA	LLE (hexane/ methyl tert-butyl ether 1:1, v/v)	• Urinary BP-conjugates were positively correlated with PBDEs in blood plasma, but were either not or negatively correlated with other organobromine compounds in blood plasma; • Stronger correlations were observed between concentrations of BDE congeners having the same number and pattern of bromine substitution on their phenyl rings in blood plasma and their corresponding BP-conjugates in urine.
	**Occupationally Exposed Workers**							
TBBPA	[32] Jakobsson (2002)	CSS CCS	Blood (serum)	Sweden 1999	**N=70** n=19 Computer technicians (15M/4F, 27–45 y) n=51 Controls	**IC for Exposed group:** occupationally exposed to flame retardants in electronic equipment **IC for Control group:** No occupationally exposed to flame retardants	LLE (hexane/ methyl tert-butyl ether 1:1, v/v)	• TBBPA levels were comparable between the computer technicians and a selected group of workers dismantling electronics, a finding that is in accordance with the short half-life of TBBPA (approximately 2 days) limiting any bioaccumulation
**Other flame retardants**								

	**General Population**							
OPFRs plasti-cizers	[40] Fromme (2014)	MCS CSS	Urine	Germany (Bavaria, Berlin, and North Rhine-Westphalia) November 2011 - May 2012	**N=312** Children (22 – 80 months)	**IC:** weight ranging from 10 to 34 kg (mean: 18 kg)	SPE LOD DCEP: 200.00 DPhP,BEP:150.00 DBP,DCEP,DCP isomers: 100.00	• For TCEP and TBEP, a significant correlations were observed between the levels in dust and the respective metabolite levels in urine; • For TCEP, there was also a significant correlation between the concentration in indoor air and metabolite levels in urine.
	**General Population**							
Dechlo-ranes	[41] Fromme (2020)	CSS	Blood (plasma)	Germany 1995, 1999, 2004, 2008, 2013-2017	**N=180** (90M/90F, 20-29 y)	NA	SPE LOQ: 0.5 pg/g lipids	• Statistically significant decrease with time was observed for DDC-DBF and DDC-Ant, but not for DDC-CO; • Medians found in blood samples in 2017 are similar to those observed in Germany in 2013/14; • Medians are higher compared to values reported in other European countries.
**Other POHs**								
	**Occupationally Exposed Workers**							
PCDDs /Fs	[25] Moon (2005)	CSS CCS	Blood (serum)	Korea 2001- 2002	**N=103** (21 - 64 y) n= 28 Workers in municipal solid waste incinerators (M) n=75 Controls (21M/54F)	**IC for Exposed group:** Working in municipal solid waste incinerators **IC for Control group:** Live for at least 3 years prior to 2002 in areas within 300 m of incinerators in large Korean cities	LLE (acetone/hexane 2:1, v/v) LOD: 0.011 pg - 0.032 pg	• Serum levels of PCDD/Fs in incinerator workers and residents near municipal solid waste incinerators in Korea were lower than those of other countries • Workers at the municipal solid waste incinerators did not have higher exposure than nearby residents

							• 1,6-HxCDF, 4-PeCDF, 1,4,6-HpCDF, 1,9-HxCDF, 1,4-HxCDD, and 1,6-HxCDD are the major congeners of PCDD/PCDFs in human serum in Korea
[42] Hansson (1997)	CSS CCS	Blood (plasma)	Sweden	**N=14** n=10 Workers in PVC-producing plant (M, 27-67) n=4 Controls	**IC for Exposed group**: PVC-producing workers **IC for Control group:** No contact with contaminated materials	LLE (chloroform/methanol 2:1, v/v) followed by SPE	• PCDDs and PCDFs are useful as markers of occupational exposure since the blood levels of these compounds reflect a subjects exposure history • 1,2,3,4,6,7,8-HpCDF, 1,2,3,4,7,8- and 1,2,3,6,7,8-HxCDFs are specific to occupational exposure in the PVC and the chloralkali industry
**General Population**							
[22] Koppen (2001)	MCS CSS	Blood	Belgium (Peer and Antwerp) June 1999 - September 1999	**N=200** (F, 50-65) n=100 from Antwerp n=100 from Peer	**IC:** • Non- or ex-smoker • Minimal living time of 10 years in the study area • Working in the town of residence or at home **EC:** Jobs with specific risks of exposure.	Soxhlet apparatus with n-hexane:acetone (2:1, v/v) LOD: 10.00-20.00	• PCDD/F body burden values in Flanders remain higher than in neighbouring countries.
[43] Galimova (2014)	CCS	Human semen	Russia (Ufa)	**N=217** n=168 Infertile men (22-41 y) n= 49 Fertile men	**IC for Infertile group:** Experienced infertility for 1–10 marriage years	LLE (hexane/diethylether/ethanol) followed by clean up	• Increased level of PCDDs/Fs in the ejaculate of infertile males compared with fertile donors suggests a rela-

						**EC for Infertile group**: Severe somatic pathology, diseases of the testes and their adnexa<br>**IC for Control group:**<br>• 1 to 3 healthy children<br>• match infertile group by age, growth and other parameters		tionship between environmental pollution and reproduction function.<br>• The detection of a typical profile of PCDDs/Fs corresponding to contemporary industrial manufacture confirms technogenic nature of sperm contamination.
	[24] Parera (2013)	MCS CSS CCS	Blood Breast milk	Spain (Mataró and Arenys de Mar) March 1995 - June 1995, 2005, 2008	**N=**258 For blood<br>n=68 Exposed from Mataró (32M/36F, 48.5 ± 15 y)<br>n=94 Controls from Mataró (47M/47F, 48.3 ± 14 y)<br>n=86 from Arenys de Mar (50M/36F, 48.2 ± 13 y)<br>n=10 Workers (7M/3F, 43.9 ±12 y)<br>**N=54** for breast milk<br>n=15 ⩽30 years old, 1st child (26.2 ± 3.4 y)<br>n=11 ⩽30 years old, 2st child n=11 (27.72 ± 3.1 y)<br>n=15 >30 years old, 1st child (34.8 ± 2.9 y)<br>n=13 >30 years old, 2st child (35.0 ± 3.3 y)	**IC for Exposed group:** Living less than 1 km from the incinerator plant<br><br>**IC for Control group:** Living more than 3 km from the incineration plant	For blood: Chromatographic glass column<br><br>For breast milk: LLE (petroleum ether, diethyl ether)	• PCDD/Fs levels in human milk are the lowest ever published;<br>• PCDD/Fs levels decrease in general population in Mataró from 1999 to 2002 and stabilization afterwards;<br>• No differences in blood levels of these compounds between subjects considered exposed and non-exposed to solid waste incineration plant were observed; main route of contamination of these compounds to humans is through other sources, mainly diet.
	**Occupationally Exposed Workers**							
Chlorophenols (CPs)	[44] Kontsas (1995)	CSS CCS	Urine	Finland	**N=52** (45M/7F)<br>n=35 Sawmill workers, n=17 Controls	**IC for Exposed group:** Workers exposed to sodium	LLE (toluene)<br>LOD:<br>2,4-DCP:3.70x10^3<br>2,6-DCP:3.10x10^3	• 2,4,6-TCP, 2,3,4,6-TeCP and PCP are detected in the sawmill worker urine samples;

						chlorophenolate product<br>**IC for Control group:**<br>• work in the sawmills,<br>• jobs had not involved contact with chlorophenols	2,4,5- TCP: 5.10 x10[3]<br>2,4,6-TCP: 4.50 x10[3]<br>2,3,4,6-TeCP: 5.40 x10[3]<br>PCP: 4.10x10[3].	• Chlorophenols were not detected in the urine from the three laboratory workers;<br>• The concentrations of CPs are either lower or of the same magnitude of which present in studies on general population or on other sawmills
**Polycyclic Aromatic Hydrocarbons (PAHs)**								
	**Occupationally Exposed Workers**							
PAHs	[51] de León-Martínez (2021)	MCS CSS	Urine	Mexico November 2019 - January 2020	**N=149** (50.05 ± 13.83 years)<br>n=42 Brickmakers (M, 55.4 ± 15.8 y)<br>n=36 Stonemasons (M, 43.8 ± 10.9 y)<br>n= 40 Indigenous workers (30M/10F, 53.8 ± 9.5 y)<br>n=31 Mercury miners (M, 45.1 ± 14.7 y)	**IC:**<br>• Older than 30 years,<br>• Living of over three years at the site of study,<br>• Be active workers in the study communities	LLE (pentane/toluene 80:20, v:v)<br>LOD/LOQ: 10.00-500.00	• High exposure to PAHs was found in people from the four activities;<br>• Of the 10 PAHs hydroxylated metabolites, 1-OH-PYR, 9-OH-FLU, 4-OH-PHE, 2-OH-NAP, and 1-OH-NAP were in higher concentrations;<br>• The indigenous population presented a different fingerprint compared to the three scenarios;<br>• The chemical fingerprints among the brickmakers and mercury mining population were similar;<br>• The concentrations were similar and in some cases higher than workers in occupations classified as carcinogenic.
	[48] Yang (2017)	CSS CCS	Urine	China (Wuhan)	**N=1472** Coke oven workers<br>n=547 Normoglycemia (463M/84F, 41.6 ± 8.1 y)	**IC:** Worked at least 1 year in a coking plant	LLE (n-hexane) followed by SPE<br>LOD: 100.00-900.00	• Elevated urinary 4-hydroxyphenanthrene was significantly associated with increased risk of diabetes;

				n =810 Impaired fasting glucose (733M/77F, 43.0 ± 8.1 y)<br>n=115 Diabetes (111M/4F, 47.6 ± 7.1 y)			• The association was more prominent in those who were smokers, overweight, with longer working years and worked at coke oven settings.
[45] Waidy-anatha (2002)	CSS CCS	Urine	China June 2000	**N=50**<br>n=28 Coke oven workers (26M/2F, 29-51 y)<br>n=22 Controls (19M/3F, 18-50 y)	**IC for Exposed group:** Occupationally exposed to PAHs<br>**IC for Control group:** No occupationally exposed to PAHs	HS-SPME<br>LOD:<br>NAP, PHE: 0.50<br>PYR: 0.30<br>BAP: 50	• Urinary levels of NAP, PHE and PYR were all associated with exposure category (controls, side- and bottom-workers, and top-workers) but not with smoking status;<br>• Strong correlations were observed between urinary levels of NAP, PHE, and PYR in coke-oven workers;<br>• Unmetabolized 2-, 3- and 4-ring PAHs can be measured in urine by HS-SPME.
[52] Wallace (2019_a)	CSS	Breath	USA (Illinois)	**N=41** Firefighters (37M/4F)	**EC:**<br>• smokers<br>• cardiovascular diseases<br>• older than 55<br>• pregnant	Automatic thermal desorption	• 60 unique exogenous chemicals and endogenous compounds, including single-ring aromatics, polynuclear aromatic hydrocarbons, volatile sulfur-containing compounds, aldehydes, alkanes, and alkenes were identified;<br>• 7 non-targeted features were found to exhibit significantly increased or decreased concentrations from pre- to post-fire suppression activity for all subjects as a group.
[46] Campo (2009)	CSS CCS	Urine	Italy	**N=15** (M)<br>n=5 Coke oven workers<br>n=5 Asphalt roofing workers<br>n=5 Controls	**IC for Exposed group:** Coke oven workers	HS-SPME<br>LOQ: 2.28 – 22. 80	• NAP and PHE are always detected, even in samples from subjects not occupationally exposed to PAHs;

					**IC for Control group:** No occupationally exposed to PAHs.		• BaA and CHR were quantifiable only in samples from coke-oven workers, while 5-ring analytes BaP, BkF and BbF were quantifiable only in two samples out of five from coke-oven workers. • Samples from coke-oven workers had the highest levels of urinary PAHs; • Levels of urinary PAHs in asphalt roofing workers were in the same range of concentration as those found in not occupationally exposed subjects.
[49] Li (2012)	CSS CCS	Urine	China (Wuhan)	**N=1287** Coke oven workers n = 496 Low exposure (428M/68F, 41.95 ± 8.35 y) n = 265 Intermediate exposure (239M/26F, 42.4 ± 7.90 y) n = 56 High exposure (56M, 42.23 ± 8.04 y) n=470 Controls (360M/110F, 42.90 ± 8.16 y)	**IC:** Occupationally exposed to PAHs **EC:** Serious medical conditions affecting heart rate variability, such as heart failure, angina, arrhythmia, myocardial infarction and other heart problems.	LLE (n-hexane) followed by SPE LOD: 100.00- 1.40x10^3	• Occupational exposure to coke oven emissions is associated with a dose-response decrease in heart rate variability; • Increased exposure to 2-hydroxynaphthalene is associated with significantly decreased heart rate variability; • Increase of working years and exposure levels has resulted in a gradual decline of heart rate variability.
[53] Smith (2012)	CSS	Urine	USA	**N=24** Air force personnel n = 6 Low exposure (4M/2F, 28.0 ± 4.2 y) n = 9 Moderate exposure (8M/1F, 25.9 ± 6.5 y) n = 9 High exposure (9M, 29.4 ± 8.5 y	**IC for Low exposed group:** Not have regular contact with jet propulsion fuel 8	LLE (pentane) LOD: 1-Naphtol: 1.22x10^3 2-Naphtol: 1.69 x10^4	• Post-shift levels of 1- and 2-naphthol increased significantly with increasing of exposure; • Post-shift levels of urinary 1- and 2-naphthol reflect JP8 exposure during the work-

							shift and may be useful surrogates of JP8 exposure.
[47] Campo (2008)	CSS	Urine	Italy	**N=10** Coke oven workers (M)	**IC:** Daily exposed to PAHs during their work.	LLE (n-hexane) followed by SPE<br>LOQ: 100.00-1.4 x10^3	• 1-hydroxynaphthalene and 2-hydroxyfluorene were the most abundant compounds in urine samples from coke-oven workers;<br>• 6-hydroxychrysene and 3-hydroxybenzo[a]pyrene were always below the quantification limit.
[50] Yang (2019)	CSS	Urine	China (Wuhan)	**N=1100** Coke oven workers<br>n=691 low risk (551M/140F, 39.5 ± 7.0 y)<br>n=409 high risk (408M/1F, 47.0 ± 6.6 y)	**IC:** Worked for at least one year in a coke-oven plant in Wuhan.<br>**EC:**<br>• Participants who had reported cardiovascular disease or cancer<br>• Profiles with missing information on components of atherosclerotic cardiovascular disease risk score or the mediator (blood lipids, systolic blood pressure, waist circumference, body mass and heart rate variability indices.	LLE (n-hexane) followed by SPE<br>LOD: 100.00- 1.40x10^3	• Urinary 1-hydroxynaphthalene, 2-hydroxynaphthalene and total PAH metabolites were dose-responsive associated with increased risk of atherosclerotic cardiovascular disease;<br>• Significant dose-dependent relationships were found across 2-hydroxynaphthalene with decreasing heart rate variability indices;<br>• Occupational exposure to PAHs may increase ASCVD risk, which was partially mediated by HRV.
**General Population**							

[14] Lu-zardo (2019)	CSS	Blood (serum)	Romania (Bra-sov) December 2017 -February 2018	**N=121**(29M/92F, 27.8 ± 9.8 y)	NA	SPE<br>LOQ: 200.00	• Phenanthrene was the most frequently detected sub-stance;<br>• Naphthalene was the PAH detected with the highest concentration;<br>• Serum levels of phenan-threne were significantly lower among younger people but the number of detected PAHs was higher in younger people from rural areas;<br>• PAH serum levels may vary between individuals with a similar exposure due to ge-netic, demographic and/or en-vironmental factors.
[55] Wirn-kor (2019)	CSS	Blood<br>Urine	Nigeria (Owerri)	**N=36** (18M/18F, 4-14 y)	NA	LLE (methylene chlo-ride/hexane)<br>LOD: 5.00-19.00	• PAHs concentrations in blood was lower than in urine;<br>• There aren't significant differences between schools, because all schools were expe-riencing similar anthropogenic disturbances;<br>• Two and three rings PAHs had generally low con-centrations in both blood and urine;<br>• The concentration of dibenzo (a,h) anthracene was highest for urine than in blood;<br>• Urine PAHs showed higher concentration of car-cinogenic PAHs than blood.
[56] Boada (2015)	CCS	Blood (serum)	Spain (Canary Is-lands)	**N=346**<br>n=140 Bladder cancer cases (120M/20F)	**IC for Bladder cancer group:**	SPE<br>LOQ 50.00	• Serum PAHs did not ap-pear to be related to bladder

	February 2007<br>-<br>August 2009	n=206 Controls (168M/38F)	• Incident bladder cancer cases<br>• Older than 18 years<br>**EC for Bladder cancer group:**<br>• Bladder cancer was secondary to another cancer<br>• Cases resided outside the CHUIMI reference area<br>• Individuals had difficulty in understanding the questionnaire due to cognitive problems<br>**IC for Control group:**<br>• Admission diagnosis unrelated to risk factors for bladder cancer or oncological diseases<br>• Admission diagnosis unrelated to known risk factors for exposure to chemical contaminants<br>• Older than 18 years		cancer risk, although the profile of contamination by PAHs was different between patients and controls;<br>• Pyrene was solely detected in controls and chrysene was exclusively detected in the cases;<br>• Phenanthrene serum levels were inversely associated with bladder cancer.

					**EC for Control group:**<br>• Previous diagnosis of any cancer<br>• History of hematuria<br>• Difficulty in understanding the questionnaire		
[57] Wang (2018)	MCS CSS	Breast milk	China (Lanzhou) April 2016 - June 2016	**N=126** Lactating women (F, range of mean age 25-30 y)<br>n=18 Xigu District<br>n=20Anning District<br>n=27 Qilihe District<br>n=33Chengguan District	**IC:**<br>• Healthy mothers<br>• Living in studied area for at least 3 years	LLE (Dichloromethane) followed by SPE<br>LOD: 0.2 to 1.6 ng/g lipids	• Qilihe District had relatively high PAHs level in human milk, followed by Xigu;<br>• Main PAHs compounds in human milk were 3–4 rings PAHs;<br>• Barbecue food could elevate the human milk PAHs levels, and frequent barbecue intake would cause 10% difference between high and low intake frequency group in our study;<br>• The difference of PAHs in human milk caused by second-hand smoke was 4–11% in this study;<br>• Ingestion doses of PAHs by infants were much higher than the inhalation doses.
[54] Zhang (2018)	CSS	Blood	China (Ningbo)	**N=14** (7M/7F, mean age 30)	**IC:**<br>• Non-smoking<br>• Non-drinking	pipette tip SPE<br>LOD: 2.00-4.00<br>LOQ:7.00-13.00	• Dominant PAHs in human whole blood were low-molecular-weight PAHs (2–4 rings);<br>• Higher-molecular-weight PAHs were found at

								relatively lower concentrations;<br>• The concentration of PAHs in blood sample ranked in the order PHE>NAP>FLU> FLT>PYR;<br>• The concentrations of 16 PAHs in female participants were relatively higher than that of male participants.
**Pesticides**								
	**Occupationally Exposed Workers**							
Pesticides	[59] Kaur (2018)	CSS CCS	Blood	India (Bathinda) 2014-2017	**N=204**<br>n=120 Pesticide-exposed workers (M, 46.1 ± 14.3)<br>n= 84 Controls (M, 36.4 ± 16.5)	**IC for Exposed group:**<br>• Healthy male persons<br>• Older than 18 years<br>• More than 3 years pesticide exposure<br>• Recent pesticide exposure of at least 2 weeks<br>• Living within 1 km range from field were included in the pesticide-exposed group<br>**IC for Control group:**<br>• Healthy male persons<br>• No history of occupational	LLE (hexane)	• Significantly elevated relative risk of lower antioxidant defence mechanism in occupationally pesticide-exposed group as compared with unexposed group;<br>• Pesticide exposure is a major risk factor leading to increased oxidative stress inside the body.

					exposure to pesticides or any kind of genotoxic environmental agent<br>**EC for Control group:**<br>• Past medical history of using drugs known to cause genotoxicity<br>• Exposure to any kind of radiations for 12 months before blood sampling,<br>• Physical injury or, behavioral disorders or mentally disturbed.		
[58] Soomro (2008)	CSS CCS	Blood (serum)	Pakistan (Sindh province)	**N=250**<br>n=140 Agriculture farmers (mean age 45.6 years)<br>n=110 Controls (mean age 44.8 years)	**IC for Exposed group:** Agriculture farmer workers exposed to pesticides<br>**IC for Control group:** Belong to farmers' community but never spray pesticides on crops or any where else.	LLE (n-hexane: diethyl ether 1:1, v/v)	• The residues in blood are likely to appear at very low concentrations because as soon as it enters into the body most of the chemical may get metabolized and the metabolites may accumulate to induce toxic effects;<br>• Ultra low quantities of the contaminants present in the body indicate toxicological impact on exposed population;<br>• Blood contamination is related to cholinesterase inhibition in the spray-workers in Sindh.
**General Population**							

[60] Cas-torina (2010)	MCS CSS	Urine	USA (Califor-nia,Salinas Valley of Mon-terey County)<br><br>September 1999 -Novem-ber 2000<br><br>1999–2002	**N=880** Pregnant women<br>n=538 CHAMACOS cohort (26.1 ± 5.2)<br>n=342 NHANES cohort (27.0 ± 5.9)	**IC:**<br>• Less than 20 weeks of gestation at the time of en-rollment,<br>• Older than 18 years<br>• Qualified to receive poverty-based government health insurance<br>• Planned to continue receiving prenatal care at a participating health center.	SPE<br>LOD:<br>2.40 – 1.30 x10^3	• Pregnant women in the Salinas Valley are chronically exposed to several current-use OP and OC pesticides and chlorinated phenols, with ad-ditional intermittent exposures to other pesticides;<br>• Pesticide metabolite de-tection frequencies were simi-lar to those found in a U.S. reference population of preg-nant women;<br>• Significantly higher 95th percentiles for several metabolites suggest that a sub-set of women experience addi-tional exposures potentially re-lated to regional agricultural pesticide use.
[61] Morgan (2015)	CSS	Urine	USA (Ohio) April 2001 - November 2001	**N=121** (10M/111F, 20 – 49 y)	NA	LLE (dichloromethane)<br>LOD: 200.00<br>TCP: 1.00 x10^3	• Most of the adults (95%) were exposed to several different pesticides over the 48-h monitoring period in Ohio;<br>• Important sociodemo-graphic or lifestyle factors (ur-banicity, employmentstatus, sampling season, 48-h sweet/saltysnack consumption, 48-h time spent outside at home, pet ownership and re-moval of shoes before enter-ingthe home) were identified that increased the exposures of the adults to these pesticides in their daily environments;

								• These factors tended to vary by pesticide.
**Organochlorine pesticides (OCPs)**								
	**Occupationally Exposed Workers**							
	[62] Ntow (2007)	MCS, CSS	Breast milk Blood (serum)	Ghana (Offinso District and Kassena-Nankana District) February 2005 - January 2006	**N=160** Farmers n=45 for milk (45F;18-40y, mean age 28y) n=115 for blood (56M; 18-53y, mean age 36y; 59F,18-74y, mean age 36)	**IC:** • Older than 18 years, • Working in vegetable farms, • Which have applied pesticides between 1 and 26 years, and are still applying pesticides	LLE (acetone/hexane 1:1, v/v) LOQ: 0.01 ng/g lipids	• The presence of persistent, bioaccumulative, and toxic DDTs, HCHs, dieldrin, and HCB in human fluids and at levels that raise public health concerns; • Almost all samples have shown detectable levels of residues of pesticides (especially p,p'-DDE).
	**General Population**							
OCPs	[21] Turci (2009)	MCS CSS	Blood (serum)	Italy (Pavia, Novafeltria)	**N=95** n=59 from Pavia (36M/23F; mean age 41.7 y) n=36 from Novafeltria (19M/17F; mean age 42.3 y)	**EC:** history of occupational or accidental exposure to PCBs and OCPs	LLE (ethyl ether/hexane 1:1, v/v) LOD/LOQ: 50.00-500.00	• A cost- and time-saving screening procedure for the determination of 16 OCPs in human serum was developed and validated; • There is a correlation between age and concentrations of p,p'-DDE, and HCB; • Gender cannot be considered as a significant variable, since no statistical difference was observed between males and females.
	[66] Kanazawa (2011)	CSS	Blood	Japan (Hokkaido) 2002 - 2005	**N=186** Pregnant women (186F, 30.8±4.8)	NA	LLE (ammonium sulfate in H_2O and 25% ethyl alcohol/hexane) LOQ: 0.6-20 pg/g lipids	• 21 chemicals are detected in pregnant women, including three chemicals with no history of use in Japan; • contamination levels of POC pesticides are low compared to previous studies and that exposure levels are decreasing with each passing year; • the body burdens of these chemicals are associated with subjects' characteristics, such as age, pre-pregnancy

							body weight(s) and history/ies of past conception(s).
[65] Botella (2004)	MCS CSS	Blood	Spain (Granada and Almeria provinces)	**N=200** Postmenopausal women undergoing surgery (F, mean age 53)<br>n=86 Malignancies<br>n=71 Bile vesicle disease<br>n=26 Abdominal or inguinal hernia<br>n=6 Varices<br>n=11 Other diseases	NA	LLE (methanol, ethyl ether/hexane 1:1, v/v)<br>LOD: 500-1.00 x10^4	• Women are currently exposed to organochlorine pesticides in Southern Spain;<br>• The frequency and level of these compounds were similar to those reported in humans in other areas of the world where many of these organochlorines have been banned.
[67] Qu (2010)	CSS	Breast milk<br>Blood<br>Cord blood	China (Guangzhou)<br>March 2004	**N=90** Young mothers	NA	LLE (hexane/dichloromethane 1:1, v/v)<br>LOD: 0.5 to 0.8 ng/g lipid	• Levels of HCH residues in human milk and maternal blood were relatively lower than those reported in other countries;<br>• The levels of DDTs were much higher than in Japan and most European countries, but significantly lower than developing countries such as eastern Romania, Slovakia, Thailand and especially India;<br>• The levels of DDT and HCH residues decreased in Guangzhou over time and also suggested that these compounds had come from the past usage.
[68] Kuang (2020)	CSS	Breast milk	China (Jinhua)<br>September 2018 - October 2019	**N=55** (F, 31.3 ± 4.6 y)	**IC:** Mother living in Jinhua city of Zhejiang Province for at least 5 years	Quechers extraction method<br>LOD: 150.00-1.27x10^4	• OPPs and PYRs were not detectable in human milk in Jinhua;

							• The detection levels of OCPs in human milk followed the order of p,p’-DDE>β-HCH> HCB;<br>• OCPs in human milk in Jinhua were generally at medium to lower levels as compared to other cities or countries;<br>• Age was positive correlated with p,p’-DDE residues in human milk;<br>• BMI, parity, dietary habit, environment, living style were not correlated to pesticide levels in human milk.
[69] Kim (2013)	CSS	Blood (serum)	Korea March 2009 - August 2009	**N=1904** (829M,1075F, 20 y – over 60)	NA	Automated HS-SPME LOD: 10.00–1.00 x10^3	• HCB was the most frequently detected OCP;<br>• p,p’-Dichlorodiphenyldichloroethylene had the highest average concentration and was detected from 7.5% of serum samples;<br>• POCPs and PDDTs concentrations increase in realtion to age;<br>• Concentration of OCPs in male serums are higher than in female serums, except in age group of the sixties.
[71] Mercado (2013)	MCS CSS	Blood (serum)	Bolivia (Algodonal, Aguas Claras, and La Junta)	**N=70** (46M/24F, >18 y)	**IC:**<br>• Older than 18 years,	LLE (acidified diethyl ether and n-hexane) followed by clean-up LOD: 100.00	• Results revealed high levels of exposure to p,p’-DDE, which might be derived from a heavily polluted local

			November 2010 - April 2011		• Living in the study area for at least 5 years.		environment and past occupational exposure;<br>• Serum p,p'-DDE concentrations were associated with time of residence in the study area, personal hygiene after work, and body mass index.
[70] Harmouche -Karaki (2018)	CSS	Blood (serum)	Lebanon (Beirut) October 2013 - June 2015	**N=314** (133M/181F, 25.6 ± 0.15)	**IC:**<br>• Lebanese nationality<br>• No medication that could affect their blood tests.	LLE (ethyl alcohol, ammonium sulfate and hexane 1:1:3,v/v) followed by clean-up<br>LOD:<br>DDT: 12.00<br>DDE 27.00<br>HCB: 22.00	• The major contributor among the four OCPs was DDE;<br>• Relatively low levels of OCPs when compared to worldwide concentrations;<br>• OCP concentrations were lower than the biomonitoring equivalents excluding any appreciable health risk;<br>• There is an inverse association between HCB concentrations and BMI as well as HCB, β-HCH, and DDE levels and smoking habits;<br>• Milk consumption however was positively associated with an increased serum level of β-HCH.
[63] Charlier (2002)	CSS	Blood	Belgium (Liège) October 2000 - August 2001	**N=251** (104M/147F, 18 – 64 y)	**IC:**<br>• Healthy individuals<br>• No occupationally expos:ed to pesticides	LLE (petroleum ether: diethylether, 98:2) followed by a SPE<br>LOD:<br>DTT: $0.50x10^3$<br>HCB: $1.00x10^3$	• Contamination has been so ubiquitous that DDT and HCB residues can still be found at detectable levels in the major proportion of the general population;<br>• Out of 251 presumably healthy individuals, only 45

								(17.93%) were free of detectable amount of organochlorine residues.<br>• The significantly most frequent residue was p,p'-DDE (66.5% of the samples) followed by HCB (13.5% of the samples).<br>• No differences between men and women were observed.
	[64] Charlier (2005)	CCS	Blood Human semen	Belgium 2001 - 2003	**N=155** for Human semen<br>n=82 Subfertile or unfertile men (26.20 ± 4.02)<br>n=73 Normofertile controls (25.10±4.39)<br>**N=42** for Blood:<br>n=23 Mothers of fertile men (54.2 ± 4.25)<br>n=19 Mothers of sub- or infertilemen (52.0 ± 6.08)	**IC for Normofertile controls:**<br>• Sperm counts higher than 20×106/ml<br>• Motility over 40%<br>• Morphologically normal spermatozoids counts higher than 14%	LLE (petroleum ether/ diethylether, 98:2) followed by a SPE<br>LOD:<br>p,p_-DDE:500.00<br>LOQ:<br>p,p_-DDE: 650	• No significant difference was found when p,p'-DDE levels were compared in blood or seminal plasma of subfertile patients and controls;<br>• There is not a significant correlation between semen quality and p,p'-DDE serum level;<br>• The mean serum concentration of p,p'-DDE was significantly higher in the group of women with subfertile sons versus those of fertile men;<br>• 100% frequency of p,p'-DDE detection in mothers of the cases was observed.
	**Occupationally Exposed Workers**							
HCB	[72] Selden (1997)	CSS CCS	Blood (plasma)	Sweden 1992	**N=27**<br>n=9 Smelter workers (M, 26-64 y)<br>n=18 Controls (M, 28-62 y)	**IC for Exposed group:** Direct involvement in the	LLE (n-hexane/diethyl ether 1:1 v/v)<br>LOD: 0.8	• A 4-fold increase of mean P-HCB was found among the exposed subjects compared with the controls

						degassing procedure<br>**IC for Control group:** Employ at the same company		and for P-OCS this difference was even larger;<br>• Within the exposed group there was a good correlation between the P-HCB and P-OCS with the cumulative number of years of exposure to hexachloroethane.
**Pyrethroids**								
	**General Population**							
Pyrethroids	[73] Wu (2013)	CSS	Urine	China (Jiangsu Province) June 2010 - January 2011	**N=481** Infants (265M/216F, 12 months)	**IC:** living in town, suburban, and countryside geographic segments in studied area.	LLE (n-hexane)<br>LOD: 100.00	• Urinary pyrethroid metabolites of 3-PBA, cis-DCCA and trans-DCCA were detectable with higher detection frequency and levels in infants;<br>• Urinary metabolites levels were comparable or slightly higher than those in the children from the other countries.
	[74] Tao (2013)	CSS	Urine	USA (Boston)	**N=10** children (3 - 11 y)	NA	auto-SPE<br>LOD:<br>metabolites: 8.00-20.00	• DCCA, 3PBA, and FPBA were detected in all urine samples;<br>• HCVA, CPBA, and DBCA were detected in seven, seven, and five urine samples, respectively;<br>• MPA was not detected in all ten urine samples.
	**General Population**							
Permethrin (3-phenoxybenzoic acid	[75] Morgan (2007)	CSS	Urine	USA (Ohio) January 2001 - November 2001	**N=127** Children (20-67 months, mean 48 months)<br>n=69 Children in the home group	NA	LLE (dichloromethane)<br>LOQ:<br>3-PBA: 400.00	• The preschool children were potentially exposed to cis- and trans-permethrin from several sources among which the primary route of

(3-PBA))					n=58 Children in daycare groups			exposure to permethrin was ingestion (dietary and indirect);<br>• The urinary biomarker of exposure to permethrin and other pyrethroids, 3-PBA, confirms that these children were exposed to and absorbed one or more of the pyrethroids, likely including permethrin, into their bodies.
**Other Pesticides**								
	**Occupationally Exposed Workers**							
GLY	[76] Zhang (2020)	CSS	Urine	China (Jiangsu and Shandong provinces)	**N=134** Workers involved in crystallization, centrifugation, drying and packaging (112M/22F, 41.1 ± 8.1 y)	**EC:**<br>• Working life less than 24 months<br>• Exposure to other pesticides during the 2 years prior to the survey and sample collection (exposure frequency >3 times a year and each exposure >8 h)<br>• Urine specific gravity <1.010 or >1.030<br>• Liver and kidney dysfunction	SPE	• The detection rates of GLY and AMPA in the urine samples were 86.6% and 81.3%, respectively;<br>• Urinary concentration of GLY and AMPA was positively correlated with the concentration of GLY in the air.

					• Administration of hepatotoxic or nephrotoxic drugs within the past 3 months		
**General Population**							
[77] Conrad (2016)	MCS CSS	Urine	Germany (Greifswald and Muenster) 2001- 2015	**N= 100** Sampling location Muenster n=80 (40M/40F, 20-28) Self-reported vegetarians/vegans (Greifswald) n=20 (5M/15F, 20-28)	**IC:** no specifically restricted diet (vegetarian or vegan)	LLE (acetonitrile, 2,2,2 − trifluoroethanol, trifluoroaceticanhydride)	• 31.8% contained glyphosate concentrations at or above the limit of quantification and AMPA in 40.1% of samples; • Quantification rates were lower again in 2014 and 2015 with32.5% and 40.0%, respectively; • Urinary glyphosate and AMPA levels tended to be higher in males.
[78] Stajnko (2020)	MCS CSS	Urine	Slovenia (Prekmurje, Goričko, Ravensko and Lendavske Gorice area) January 2018 - March 2018 and May 2018 - June 2018	**N=246** Children and adolescents n=149 Children (67M/82F, 9.0 ± 1.0 y) n=97 Adolescents (54M/43F, 14.0 ± 0.8 y)	**IC:** • Living in one of the selected areas • Continuous living in one of the selected areas for at least 3 years • Age between 7 and 10 years, or between 12 and 15 years, • No intake of medication due to chronic	LLE (acetonitrile, 2,2,2 − trifluoroethanol, trifluoroaceticanhydride)	• There is very low exposure to GLY and its metabolite AMPA in children and adolescents living in an agricultural environment in the northeastern part of Slovenia; • GLY and AMPA were detected in 27% and 50% of urine samples from the first sampling period, respectively; and in 22% and 56% from the second sampling period, respectively. • Children rather than adolescents tended to have higher exposure, as did, boys

						metabolic diseases		rather than girls among adolescents;<br>• The stronger AMPA and GLY correlations and the correlations with elements observed in the second sampling period might be connected to the more intensive use of glyphosate based herbicides in the spring.<br>• At such low exposure concentrations, age and gender were confirmed as determinants of the GLY and/or AMPA exposure
**Other POPs**								
**General Population**								
PFASs	[28] Sochorová (2017)	CSS	Blood (serum)	Czech Republic (Prague, Ostrava, Liberec and ˇZd'árnad Sázavou)<br><br>2015	**N=300** (171M/129F, 18 -65 y)	**IC:** Living in the studied area for at least 3 years	SPE<br>LOQ:<br>10.00-300.00	• The frequent occurrence of PFASs in the Czech adult population is high, with PFOA,PFNA, PFDA, PFUdA, PFHxS and PFOS that are the most abundant;<br>• PFOS concentrations were the highest.;<br>• Levels of PFAs in the Czech Republic are relatively lower than those reported in other studies worldwide.

Table S2. Summary of included studies concerning Volatile Organic Compounds (VOCs).

Pollutant abbreviation	First Author (Years)	Study design	Biological Fluid	Country (Region or City) and Sampling period	Population and group characteristics [N, type, n, (sex, age)]	Inclusion (IC)/ exclusion (EC) criteria	Extraction methods (Solvent and ratio) and Limit of detection (LOD) and/or quantification (LOQ) (ng/L)	Main findings
	**Occupationally Exposed Workers**							
VOCs	[84] Ceballos (2019)	CSS	Blood Breath	USA (Boston) November 2016 - June 2017	**N=10** Nail technicians (10F, 45.0 ±10.8 y)	**IC:** • Female, • Non-smoker, • Older than 18 years • Working full-time (at least 35 hours a week) in a nail salon.	HS-SPME LOD: Breath: 0.02-0.16 Blood: NA	• Blood levels were significantly higher post-shift than pre-shift for toluene and ethyl acetate; • Methacrylates were not measured in blood and breath because of their instability.
	[82] Jalali (2016)	CCS	Breath	Iran March 2014 - October 2014	**N=44** n=20 Workers exposed to crystalline silica dust (M, 42.7 ± 8.5) n=4 Positive controls (51.0 ± 4.9) n=20 Negative controls (41.4 ± 6.9)	**IC for Exposed group:** Five year working experience or more in 2 casting workshop **IC for Negative control group:** • Healthy volunteer • Employ in office work • No occupational exposure to dust	HS-SPME	• Forty VOCs were found in all exhaled breath samples; • Concentrations of acetaldehyde, hexanal, nonanal, decane, pentadecane, 2-propanol and 3-hydroxy-2-butanone were higher in exhaled breath of the workers exposed to silica and silicosis patient compared to the healthy smoker and nonsmoker controls.

				• No history of asthma and lung disease in the past 24 months		
[85] Cheng (2019)	CSS	Breath	Taiwan	**N=13** Operating room personnel (6M/7F, 37.3 ± 7.4)	**IC:** Occupationally exposed to VOCs	Active SPME trap, a cold dehydration trap and a cold trap LOD: 0.2 ppb	• There were 18 volatile organic compounds identified in the samples; • The average concentrations of sevoflurane, dimethyl sulphide and methyl methacrylate in breath samples collected after surgical operations were significantly higher compared to those obtained before surgical operations; • There was only slight elevations were present for benzene and hexamethyldisiloxane.
[83] Erb (2019)	CSS	Urine	France	**N=63** Workers in industries that use aromatic and chlorinated VOCs n=29 Paint production plant n=34 Oil refinery	**IC:** Exposed workers to VOCs	Dynamic headspace sampler LOD: 5.00	• The difference between controls and BTEX-exposed workers was significant; • With the exception of styrene, chloroform, dichloromethane and toluene, the VOCs studied were detected and measurable in all pre-shift urine samples; • In post-shift urinary samples, more quantifiable levels of VOCs were measured with significantly higher medians for ethylbenzene and toluene.
[81] Wallace (2019_ b)	CSS	Breath	USA (Illinois)	**N=41** Firefighters (37M/4F, 18 - 55 y)	**IC:** • (Firefighters' which are exposed to VOCs • No cardiovascular disease,	Thermal desorption LOD: 0.5 ppb	• Benzene concentrations were found to be elevated post-exposure in both fire attack, victim search, and outside ventilation firefighting positions; • Certain firefighters may be more susceptible to post-exposure

				• No tobacco use,<br>• No eating char-grilled and smoked foods for 24 hr prior to the start of the study.		volatile organic compounds increases than others.
[80] Fent (2015)	CSS	Breath	USA (Chicago)	**N=6** Firefighters	**IC:** occupationally exposed to BTEX	Thermal desorption LOD 700.00-1.00x10^3	• Compared to pre-burn levels, breath concentrations of benzene, toluene, and styrene after the burns increases in mean of >2 fold;<br>• Air concentrations of BTEXS measured offgassing from firefighters' used PPE and in firefighters' postburn exhaled breath were significantly correlated.
**General Population**							
[89] Ashley (1994)	CSS	Blood	USA	**N=600 or more** people who participated in the Third National Health and Nutrition Examination Survey	**IC:** Non occupationally exposed to VOCs	Purge and trap	• There are detectable concentrations of 1,1,1-trichloroethane, 1,4-dichlorobenzene, 2-butanone, acetone, benzene, chloroform, ethylbenzene, m,p-xylene, styrene, tetrachloroethene, and toluene in most of the blood samples of non-occupationally exposed people.
[90] Jia (2012)	CSS	Blood	USA 1999 - 2000	**N= 364** (20-59 y)	NA	HS-SPME LOD: 0.29 - 7.10	• Median blood/air distribution coefficients (popKs) ranged from 3.1 (MTBE) to 77.3 (p-dichlorobenzene) in non-smoking adults;<br>• Smoking elevated popKs by 1.5–3.5 times for aromatic compounds, but did not affect the

							popKs for methyl tert-butyl ether (MTBE) or chlorinated compounds.<br>• Drinking water concentration was a modifier of MTBE's popK.
[95] Longo (2018_a)	CSS	Human semen	Italy (Lecce)	**N=57** (57M, reproductive-age)	NA	HS-SPME	• Forty-nine total VOCs are detected;<br>• 43% of VOCs are present only in one individual, while the remaining 57% in at least two semen samples;<br>• 1H, 1,2,4-Triazole is present exclusively in severe asthenozoospermic samples and Butanal, 3-methyl and Butanal, 2-methyl- are more concentrated in samples with lower motility.
[79] Uddin (2014)	MCS CSS	Blood	USA (Louisiana) May 2002	**N=297** participants (15-91 y) n=204 Calcasieu Parish participants (82M/110F) n=93 Lafayette Parish participants (40M/51F)	**IC for Calcasieu Parish group:**<br>• Non-smokers<br>• Living in Calcasieu Parish in the 5 years before the study<br>• Older than 15 years.<br>**IC for Lafayette Parish group:**<br>• Living in Lafayette Parish in the 5 years before the study<br>• Older than 15 years.<br>**EC:** Past residence in Calcasieu Parish.	HS-SPME LOD: 20.00-92.00	• VOC levels in these two populations were also compared at the national levels;<br>• Levels of 30 VOCs were measured in blood samples;<br>• The frequency of detection was >95% for benzene and m-/p-xylene, >60% for 1,4-dichlorbenzene and toluene, 27% for ethylbenzene, and 39% for styrene.

	[97] Longo (2019)	CSS	Human semen Blood Urine	Italy (Campania Region)	(M, 18 y)	**IC:** Young men	HS-SPME	• VOC composition in the human semen was richer than that of the other biosamples (urine, blood and hairs);<br>• VOC fingerprinting is able to classified the different biological fluids.
	[96] Longo (2018_b)	CSS	Human semen	Italy (Lecce)	**N=69** Men with fertility problems (M, 37 ± 5 y)<br>n=28 Severe asthenozoospermic<br>n=19 Asthenozoospermic<br>n=22 normozoospermic	**IC:** Men with inferility problems	HS-SPME	• 196 different VOCs were detected in human semen samples and 70 VOCs was detected in at least two samples;<br>• The three most recurring compounds in the samples were pyrrole, ethanol and 2-methyl-butanal;<br>• 2-Methyl-1-Pentene and Propylyclohexane are exclusive of subjects with high motility sperm.<br>• 3-Methylbutyl acetate and 1-Ethoxyethene are specific<br>• for subjects with slight asthenozoospermia,whereas O-Pentylhydroxylamine and 2, 6-Dimethylpyrazine for severe asthenozoospermic samples.
	[94] Delfino (2003)	CSS	Breath	USA (California) November 1999 - January 2000	**N=21** Children with asthma (17M/4F, 10 – 16 y)	**IC:**<br>• A minimum 1 year history of physician-diagnosed asthma,<br>• Age 10–15 years<br>• Nonsmokers who live in nonsmoking households	Autosampler LOD: 0.1 ppb	• Eight VOCs were quantifiable in >75% of breath samples (benzene, methylene chloride, styrene, tetrachloroethylene, toluene, m,p-xylene, o-xylene, and p-dichlorobenzene);<br>• There is a positive associations between bothersome or more severe asthma symptoms and same day breath concentrations of benzene but not other breath VOCs;

				• Home address and school address within a 3 mile radius of the central air monitoring site<br>• At least two symptomatic days per week requiring as needed β-agonist inhaler use		• Ambient petroleum-related VOCs measured on the same person-days as breath VOCs showed notably stronger associations with symptoms, including toluene, m,p-xylene, o-xylene, and benzene.
[91] Lee (2010)	CSS Urine	Korea	**N=100** Partecipants in the *Herbei Spirit* oil spill cleanup work (87M/13F, 20.7 ± 2.1 y)	**EC:** Occupationally exposed to VOCs	SPE LOD: $3.00x10^3$-$10.00x10^3$ LOQ: $10.00x10^3$-$30.00x10^3$	• The VOC metabolites, except hippuric acid, were present at higher levels in the urine samples of volunteers after cleanup work;<br>• The levels of mandelic acid and trans,trans-muconic acid were increased significantly;<br>• The exposure effect was greater in women than in men;<br>• The effect of smoking was analysed in all exposed and non-exposed groups, with non-smokers showing increased MA and t,t-MU levels related to exposure.
[92] O'Lenick (2018)	CSS Urine	USA (North Carolina) November 2009 - May 2011	**N=28** (15M/13F, 31.1 ± 7.2)	**IC:**<br>• Healthy adults<br>• Aged between 18 to 50 years<br>• Not pregnant<br>• No occupational exposure to pyrethroid insecticides,	Thermal desorption LOD: 24.00 - $8.34x10^3$	• 28 polar VOCs (PVOCs) detected in human urine;<br>• 2-Pentanone, 4-heptanone, 2-butanone, and 4-methyl-2-pentanone have all been found consistently in urine from healthy individuals;<br>• There are metabolites of phthalates in urine samples.

					• No pre-existing medical conditions that would affect urine output (i.e., kidney, liver, or heart disease) • Currently living in a residential home or apartment • Speak, read, and use English fluently		
	[93] Wang (2007)	CSS Urine	Japan 2003	**N=24** (14M/10F, 32 ± 15 y)	**IC:** Subject's house had to be built no more than 5 years before the year of study	Automatic headspace sampler LOD: 10.00	• Toluene, ethylbenzene, xylene isomers, styrene and p-dichlorobenzene are detected in urine samples; • There are significant correlations between the time-weighted average air concentrations in the bedroom and morning urinary concentrations for toluene, o-xylene, total xylene and p-dichlorobenzene.
**Volatile Halogenated Hydrocarbons**							
	**General Population**						
VHH	[98] Barkley (1980)	CSS Blood Urine Breath	USA (New York) July 1978	**N=9**	NA	Thermal desorption	• 18 halogenated compounds are detected in human breath; • 9 halogenated compounds are detected in blood and urine samples; • There is not statistical correlation between exposure levels and body burden levels.
DCB	**Occupationally Exposed Workers**						

Azo dyes	[99] Guer-bet (2007)	CSS	Urine	France (Rouen) August 2003-Dicem-ber 2003	**N=47** Chemical plant workers (M, 38.9±11.3 y)	**IC:** Occupationally exposed to DCB	SPE LOD: 500.00	• Dichlorobenzidine traces ranging from 1.6 to 8.9 ppb; • DCB was detected in 4 (8%) samples of the exposed as well as in 4 (8%) samples of non-exposed workers.
	**Occupationally Exposed Workers**							
OCS	[72] Selden (1997)	CSS CCS	Blood (plas ma)	Swe-den 1992	**N=27** n=9 Smelter workers (M, 26-64 y) n= 18 controls (M, 28-62 y)	**IC for Exposed group:** Direct in-volvement in the degassing proce-dure **IC for Control group:** Employ at the same company	LLE (n-hex-ane/diethyl ether 1:1) LOD: 0.6	• P-OCS difference among the exposed subjects compared with the controls was very larger; • Within the exposed group there was a good correlation be-tween P-OCS with the cumula-tive number of years of exposure to hexachloroethane.
**BTEX (Benzene-Toluene-Ethylbenzene-Xylene)**								
	**Occupationally Exposed Workers**							
BTEX	[100] Rafiee (2019)	CSS CCS	Urine	Iran (Teh-ran) May 2018	**N=45** n=25 Workers of composting facilities (M, 37 ± 2.42 y) n=20 Controls (M, 33 ± 2.57 y)	**IC**: Healthy partici-pants without any specific disease or disorders **IC for Exposed group**: workers of composting facili-ties **IC for Control group**: no occupa-tionally exposed to BTEX	HS-SPME LOD: B: 3.20 T: 3.00 E: 1.70 o-X: 19.60 m + p-X: 7.70 LOQ: B: 10.70 T: 10.00 E: 6.70 o-X: 64.60 m + p-X: 24.70	• The geometric mean levels of urinary benzene, toluene, ethylbenzene, m-p xylene, and o-xylene in the exposed subjects were 1.4 to 3.7-time higher than values in control group; • Post-shift levels were sig-nificantly higher than pre-shift for all chemicals; • Significant associations were seen between urinary BTEX and smoking, ETS exposure, and Ramadan fasting; • Use of personal protective equipment did not reduce urinary levels.

[101] Rafiee (2018)	CSS CCS	Urine	Iran (Tehran) April 2017 - June 2017	**N=45** n=25 Waste autoclave operators (M, 37 ± 2.42) n=20 Controls (M, 33 ± 2.57)	**IC for Exposed group**: occupational exposure to BTEX in healthcare waste autoclave operators **IC for Control group:** no BTEX exposure	HS-SPME LOD: B: 3.20 T: 3.00 E: 1.70 o-X: 19.60 m + p-X: 7.70<br><br>LOQ: B: 10.70 T: 10.00 E: 6.70 o-X: 64.60 m + p-X: 24.70	• With the exception of ethylbenzene, subjects in the exposed group had significantly higher urinary BTEX levels than control group;<br>• Urinary BTEX concentrations in the exposed case group were 2.5-fold higher than in the control group;<br>• There was a significant relationship between the amount of generated waste per day and the urinary BTEX in the exposed group.<br>• Smoking status and type of autoclave used were also identified as predictors of urinary BTEX concentrations<br>• The appropriate personal protection equipment and control measures capable in reducing BTEX exposure should be provided to HCW workers to reduce their exposures to BTEX.
				**General Population**			
[102] Bergamaschi (1999)	CSS	Urine Blood	Italy December 1997 - February 1998	**N=24** Cyclists (12M/12F, 25–37 y)	**IC:**<br>• Non-smokers,<br>• Commonly using the bicycle to move within the city area	SPME	• As compared to pre-run values, benzene and toluene in blood, and toluene and xylenes in urine significantly increased after urban runs;<br>• Even at very low exposure levels, a 2-h bike run in a polluted urban environment may give rise to measurable changes in biomarkers of internal dose of selected aromatic hydrocarbons.

	[104] Hajimiragha (1989)	CSS CCS	Blood	Germany (Düsseldorf and surrounding areas)	**N=27** n = 13 Non-smokers n = 14 Smokers	**IC:** • Live in a large urban agglomeration in the Federal Republic of Germany • No occupational or hobby-related exposure to volatile organic hydrocarbons	Thermo-desorption LOD: B: 21.00 T: 80.00 E: 28.00 m + p-X: 152.00 o-X: 50.00	• BTEX were detected in measurable amounts in all blood samples, reflecting the ubiquitous exposure of humans to these agents in the urban environment; • Smokers were found to have significantly higher blood concentrations of benzene and toluene than nonsmokers; • The concentrations of ethylbenzene and xylenes also tended to be higher in smokers when compared to non-smokers. • The different concentrations of these compounds in the blood of non-smokers appear to reflect the common concentration pattern found in outdoor urban air as well as in indoor air.
	[105] Lee (2019)	CSS	Urine	Korea	**N=20** Children (4–15 years)	**IC:** • No exposure history to BTEX • No health problems	Automated HS-SPME LOD:20.00-150.00 LOQ 50.00 – 550.00	• The specific biomarkers for toluene and ethylbenzene, o-cresol and 2-ethylphenol, were detectable in all urine samples and ranged from 0.4 to 13.8 µg/L and 0.13 to 4.26 µg/L, respectively. • The specific biomarkers of m-xylene and p-xylene, 3-methylbenzylalcohol and 4-methylbenzylalcohol were detected in almost all urine samples, and the detected concentration ranges were 1.96–38.0 µg/L and 0.59–29.4 µg/L, respectively.
	**Occupationally Exposed Workers**							
Benzene	[106] Menezes	CSS	Breath	Brazil	**N=45** (M) n=15 Workers exposed to low	**IC:** • Workers exposed to benzene,	SPME LOD: 0.24 LOQ: 0.80	• The correlations between benzene concentration in ambient

(2009)			(Belo Horizonte)	levels of benzene n=30 Workers directly exposed to benzene	• Non-smokers.		air and in expired air showed a significant tendency to linearity.
[103] Campo (2011)	CSS	Urine	Italy (Milan) 2004	**N=130** Urban traffic policemen (77M/53F, 24 – 47 y)	**IC:** Occupationally exposed to MTBE	HS-SPME LOQ 10.00	• Median benzene urinary level in end-of-shift samples was 207 ng/L (BEN-U); • BEN-U was influenced by the month and smoking habit, and positively correlated to urinary creatinine; moreover, an interaction between CO and smoking was found; • BEN-U is determined mainly by personal characteristics.
[108] Qu (2000)	CSS CCS	Urine	China (Tianjin) .	**N=61** n=25 Glue- and shoe-making factories workers (11M/14F, 32.1 ± 9.0 y) n=25 Controls (12M/13F, 31.7 ± 9.3 y) n=11 Glue factory workers (4M/7F)	**IC** for Exposed group: occupational benzene exposure **IC** for Control group: no benzene exposure	NA	• Levels of benzene metabolites measured in urine samples collected from exposed workers at the end of workshift were significantly higher than those measured in unexposed subjects; • The large increases in urinary metabolites from before to after work strongly correlated with benzene exposure; • Concentrations of these metabolites in urine samples collected from exposed workers before work were also significantly higher than those from unexposed subjects.
**General Population**							
[107] Wester	CSS	Breath	USA	**N=30** (M, >18 y)	**IC:**	Silica trap LOD: 100.00	• Benzene in smokers' breath was greater than in nonsmokers'

	(2009)			(San Francisco and Stinson Beach, California)		• Older than 18 years,<br>• Residence in the city of San Francisco		breath and smokers' ambient air in both areas;<br>• Benzene in SF nonsmokers' breath was greater than in SB nonsmokers' breath;<br>• Marijuana-only smokers had benzene breath levels between those of smokers and nonsmokers.<br>• There was little correlation between benzene in breath and number of cigarettes smoked, or with other benzene exposures such as diet.<br>• Of special interest was the finding that benzene in breath of SF and SB nonsmokers was greater than that in nonsmokers ambient air. This suggests an additional source of benzene other than outdoor ambient air.
**Other VOCs**								
	**General Population**							
Acrolein	[109] Dias (2017)	CSS	Breath	Brazil (Belo Horizonte) June 2015 - July 2015	**N=30** (13M/17F, 20-55y)	**IC:** Exposed to cigarette smoke	SPME with cold fiber LOD 2.40 LOQ 3.79	• A good Pearson correlation coefficient was observed between the concentration of acrolein in indoor air and exhaled air, allowing to propose acrolein breath as environmental exposure biomarker.
	**Occupationally Exposed Workers**							
MTBE	[103] Campo (2011)	CSS	Urine	Italy (Milan) 2004	**N=130** Urban traffic policemen (77M/53F, 24–47 y)	**IC:** Occupationally exposed to MTBE	HS-SPME LOQ 10.00	• Median MTBE urinary level in end-of-shift samples was 147 ng/L;<br>• MTBE-U was influenced by the month of sampling and

								positively correlated to the time spent in traffic guarding, CO exposure and atmospheric pressure, while negatively correlated to wind speed;<br>• MTBE-U is a reliable marker for assessing urban traffic exposure.
	**Occupationally Exposed Workers**							
Anaesthetics<br><br>Sevoflurane (SEV)<br>Isoflurane (ISO)<br>Halothane<br>Isopropyl alcohol (IPA)<br>N_2O	[86] Al-Ghanem (2008)	CSS	Urine	Jordan	**N=18** Operating room personnel	**IC:** Occupationally exposed to volatile anaesthetics	HS autosampler	• Values of the post-shift urine samples were 1234±209 µg/l, 4.3±0.82 µg/l, 3.75±0.7 µg/l and 9.9±1.2 µg/l for nitrous oxide, sevoflurane, isoflurane and halothane, respectively;<br>• N_2O e sevoflurane levels are very high in post-shift urine samples;<br>• The levels of anaesthetic agents found are generally exceeding the international recommended exposure limits.
	[88] Ghimenti (2015)	CSS	Breath	Italy (Pisa)	**N=14** Hospital staff<br>For SEV: n=5 Anaesthesiologists (2M/3F, 29 ± 2 y)<br>For IPA: n= 9 Nurses(3M/6F, 29 ± 9 y)	**IC:** Occupationally exposed to anaesthetics	Thermal desorption<br>LOD: $13x10^3$	• SEV and HFIP were present in all subjects at concentrations in the range of 0.7–18, and 0.002–0.024 ppbv for SEV and HFIP respectively;<br>• Correlation between IPA ambient air and breath concentration confirmed the inhalation pathway of exposure and breath-borne IPA was measured as high as 1500 ppbv.<br>• Although the overall exposures documented were generally below levels of health concern in this limited study, outliers were

							observed that indicate potential for acute exposures.
	[87] Accorsi (2003)	CSS Urine CCS	Italy	**N=134** n=124 Operating room personnel (43M/81F) n=10 Controls	**IC for Exposed group:** Occupationally exposed to anaesthetics **IC for Control group:** No exposed to anaesthetics	Headspace autosampler LOD SEV: 30.00 N_2O: 300.00 LOQ SEV: 100 N_2O: 1.00x 10^3	• Median (range) post-shift urinary values were 1.2 µg/l urine (0.1 ‑ 5.0) for sevoflurane and 10.9 µg/l urine (0.5 ‑ 74.9) for N2O; • Urinary levels closely correlated with environmental data.
	**General Population**						
n-Alkanes	[110] Chambers (2008)	CSS Blood	USA	**N=1200**	**IC:** No occupational exposure to fuels	HS-SPME LOQ: 89.00-250.00	• A new method for the analysis of alkanes (n-hexane, n-heptane, n-octane, n-nonane, n-decane, n-undecane, and n-dodecane) has been developed; • Median blood levels for all n-alkanes were below these quantification limits; • n-Hexane levels above the method detection limit were, however, found in 1.3% of the samples.
	**Occupationally Exposed Workers**						
Cyclophosphamide	[111] Sugiura (2010)	CSS Urine	Japan	**N=41** Hospital staff	**IC:** Occupationally exposed to cyclophosphamide	LLE (ethyl acetate) LOD: 0.1 x10^3	• In the urine collected from the staff of D University Hospital, no CP was detected. • From the staff of A University Hospital, B Corporate Hospital and C Corporate Hospital, on the other hand, CP was detected at high levels. • The urinary excretion of CP per subject was between 2.7 and 462.8 ng/24 h.

								• The range of urinary excretion for each hospital was between 4.6 and 211.2 ng/24 h.

Table S3. Summary of included studies concerning pollutants different from POPs and VOCs.

Pollutant Abbreviation	First Author (Years)	Study Design	Biological Fluid	Country (Region or City) Sampling Period	Population and Group Characteristics [N, Type, n, (Sex, Age)]	Inclusion (IC)/ Exclusion (EC) Criteria	Extraction Methods (Solvent and Ratio) and Limit of Detection (LOD) and/or Quantification (LOQ) (ng/L)	Main Findings
	**Occupationally Exposed Workers**							
Diisocyanates	[125] Rosenberg (2002)	CSS CCS	Urine	Finland	**N=31**<br>n=21 Workers exposed to the thermal degradation products of polyurethanes<br>n=10 Controls	**IC:** Occupationally exposed to polyurethanes	LLE (toluene)<br>LOQ<br>Ar. diamines: 0.075-0.22<br>Al. diamines: 2.37	• TDA and MDA were detected in urine samples from workers in car repair shops and MDA in samples from workers welding district heating pipes.<br>• The 2,4-TDA isomer accounted for about 80% of the total TDA detected.<br>• No 2,6-TDA was found in the urine of non-exposed workers.<br>• The highest measured urinary TDA and MDA concentrations were 0.79 nmol $mmol^{-1}$ creatinine and 3.1 nmol $mmol^{-1}$ creatinine, respectively.<br>• The concentrations found among non-exposed workers were 0.08 nmol $mmol^{-1}$ creatinine for TDA and 0.05 nmol $mmol^{-1}$ creatinine for MDA (arithmetic means)
	**Occupationally Exposed Workers**							
Phthalates	[126] González (2019)	CSS	Blood Urine	Spain (Constantí and Tarragona)	**N=29** Workers in hazardous waste incinerator (18M/11F)	**Exposed group IC:** occupational exposure to organic substances	DLLME (Acetonitrile, tetrachloroethylene, acetic anhydride).<br>LOQ: 0.05–0.15	• Among the 8 BP analogues assessed, BPA presented the highest levels

					and heavy metals in waste incinerator		in both biological samples, with mean total (free + conjugated) BPA concentrations of 0.58 and 0.86 µg/L in blood and urine, respectively.<br>• Free vs. total BPA levels presented a mean percentage of 79% in blood and 19% in urine.<br>• Beyond BPA, traces of BPB were also found in a single sample of blood.<br>• None of the remaining BP analogues was detected in blood or urine
**General Population**							
[127] Pednekar (2018)	CCS	Blood (plasma)	India (Mumbai) May 2015 – August 2015	**N=79**<br>n=45 Infertile women (28.4±4.4)<br>n=34 Fertile women (26.8±3.8)	**IC for Infertile group:**<br>• Age between 20 and 40 years<br>• Attending infertility outpatient department<br>• Diagnosed with infertility (endometriosis, polycystic ovary syndrome)<br>• Male factor is normal<br>**IC for Fertile group:**<br>• Married women between 20 and 40 years<br>• Proven fertility<br>• No evidence of any gynaecological disorders	SPE and derivatization procedure<br>LOQ:<br>BPA,MEHP, MEHHP: 1.00<br><br>MMP, MBzP:5.00	• BPA was detectable in 77 per cent of plasma samples of infertile women and 29 per cent of fertile women.<br>• All the four phthalate metabolites namely mono-methyl phthalate (MMP), mono-benzyl phthalate (MBzP), mono-2-ethylhexyl phthalate (MEHP) and mono-(2-ethyl-5-hydroxyhexyl) phthalate (MEHHP)]were detected in plasma samples of both fertile and infertile women.

						• Achieved pregnancy naturally and delivered recently (within one year).<br>**EC for both groups:** Women on prolonged hormonal medications<br>**IC for both groups:**<br>• Live in urban areas<br>• No history of any occupational exposure to reproductive toxicants<br>• Non-smokers<br>• Not consuming alcohol		
	**Occupationally Exposed Workers**							
Hexahydro phtalic anhydride<br><br>HHPA	[128] Jonsonn (1990)	CSS<br>CCS	Urine	Sweden	**N=13**<br>n=5 Workers exposed to hexahydrophtalic anhydride (M, 24-61 y)<br>n=8 Controls (M, 28 – 50 y)	**IC for Exposed group:** Workers exposed to epoxy resins<br><br>**IC for Control group**: No known exposure to HHPA.	LLE<br>(ethyl acetate)<br>LOD: $4x10^4$	• The mean level of HHP acid in post-shift urine samples from exposed workers was 1.6 (range, 0.9-2.8) μmol/mmol creatinine;<br>• The controls showed < 0.1 mol HHP acid/mmol creatinine;<br>• There are statistically significant differences between the levels of HHP acid in post-shift vs control samples and between the values obtained for post-shift vs morning specimens from the workers;

								• There is an association between time-weighted levels of HHPA in air and of HHP acid in post-shift urine samples.
	**Occupationally Exposed Workers**							
N-methyl-2-pyrrolidone NMP	[129] Bader (2006)	CSS CCS	Urine	Germany	**N=10** n=7 Workers an adhesive bonding compounds and glue production facility n=3 controls	**IC:** Occupationally exposed to NMP	LLE (12 M potassium hydroxide/toluene 2:1, v/v) LOD: $1.00x10^4$	• NMP and its metabolites were detected in two pre-shift urine specimens; • NMP and 5-HNMP concentrations in post-shift urine samples of five workers and three on-site study examiners were below 125 µg/g creatinine and 15 mg/g creatinine, respectively, while two vessel-cleaning workers showed significantly higher urinary NMP concentrations of 472 and 711 µg/g creatinine and 5-HNMP concentrations of 33.5 and 124 mg/g creatinine. • 2-HMSI was detectable in four post-shift samples (range: 1.6–14.7 mg/g creatinine). • The vessel cleaner with the highest NMP exposure reported irritation of the eyes, the upper respiratory tract and headaches
	**General Population**							
Parabens Methylparaben Ethylparaben Propylparaben Butylparaben	[130] Feizabadi (2020)	CSS	Urine	Iran (Isfahan)	**N=100** (50M/50F, 12 – 20 y)	**IC:** • No history of chronic illness • Not taking long-term medication • Living in the study area for more than 1 year • Having a minimum age of 12 years and up to 20 years	DLLME (chlorobenzene, acetone) LOD: MP: 14.00 EP: 15.00 PP: 16.00 BP: 46.00 LOQ: MP,EP,PP:50.00 BP: 150.00	• Median concentrations of MP, EP, PP, and BP were 92.21, 8.46, 12.26, and 8.42 µg/g creatinine, respectively. • There was a strong positive significant correlation between MP and PP and moderate to a weak correlation between the other parabens. • The concentration of urinary MP in females was significantly higher than those in male. • There was a significant negative association between different BMI groups and MP and EP.

	• Completing all necessary information in relevant questionnaires	• There also was a positive significant association between the MP and age, and between MP, EP, and PP, and tobacco use.<br>• Although the estimated daily intake of the parabens was lower than the Acceptable Daily Intake, it was higher than those reported in other countries

Table S4. Summary of included studies in which gas sensors are used for VOC detection.

VOC Name	First Author (Years)	Materials	Type of Devices (Operation Principles)	Application	Main Findings
Acetaldehyde, Acetone, Ethanol, Ethyl acetate, Isoprene and n-pentane	[113] Jaeschke (2020)	Commercial semiconducting MOX	eNose-based analyser contains three sensing arrays of commercially available semiconducting MOX gas sensors: one compartment contains 8 analog MOX sensors, while the other two compartments comprise 10 digital MOX sensors	VOCs of interest in breath diagnostics under high humidity conditions, which characterize real breath matrices	• The presented instrument is able to discriminate between mid-range (3–18 ppm) concentrations of different volatile organic compounds (VOCs).

Acetone, Ethanol, Acetic acid, Formaldehyde and gas mixing	[116] Liu (2017)	Interdigital electrodes modified with graphene, ZnO and nitro-cellulose membrane	Smartphone-based sensing system for real-time VOC monitoring using alternative current impedance measurement	VOCs in exhaled breath and possibly allowed for early diagnosis of some diseases	• The smartphone-based system could detect acetone as low as 1.56 ppm in real time by combining hand-held impedance monitoring device and smartphone; • The selectivity of the system was also tested by characteristic frequencies of VOCs.
Methanol, Ethanol, Acetone and Ammonia	[115] Eambaipreuk (2011)	Metal-porphyrin sensors	Optical Electronic Nose System Setup	Exhaled breath based on different activities. Therefore, this optical e-nose system can be applied for diagnosis of health status based on breath analysis	• Principal component analysis clearly discriminates acetone (5% aq.), ammonia (5%, 10%, and 15% aq.) and acetone (2.5% aq.) mixed ammonia (2.5% aq.); • This e-nose system clearly distinguishes exhaled breath based on different activities.
Fecal VOCs	[124] Berkhout (2016)	Unique non-conducting polymer coating, containing conductive carbon black material	eNose Cyranose320®	Assess the effects of environmental factors, the influence of sampling conditions and influence of sampling method on fecal VOC composition	• A broad range of sampling conditions and environmental factors seem to have a significant influence on detected fecal VOC profiles, as measured by an eNose device; • Analysis of fecal VOCs may hold promise as a diagnostic tool, although different methodological challenges need to be overcome.
Acetone	[12] Jayasree (2014)	3 sensors of TGS822 sensing element is a tin dioxide (SnO2) semiconductor	Array of MOX gas sensors	A prototype model was developed to detect the presence of acetone in the breath, which is a biomarker for diabetes mellitus	• TGS 822 is a very suitable sensor for the detection of acetone in breath which could be a non-invasive technique for the proof of identity of diabetes mellitus; • This study has been helpful in designing an electronic nose for detecting other vapours like benzene, ammonia and Nitric oxide.

Polar VOCs (acetone, ethanol, chloroform) and nonpolar VOCs (n-hexane, toluene, isoprene)	[119] Kus (2021)	Gold nanorod (AuNR) and silver nanocube (AgNC) molecules were functionalized with thiol (S- terminal) containing Calix[4]arene	Surface acoustic wave (SAW) transducer device	Detection of markers for disease diagnosis, such as toluene present in exhaled breath of lung cancer patients	• After AuNR and AgNC modification with Calix[4]arene, sensitivity of sensors has reached up to six to eightfold higher than the individual responses, with a selectivity towards chloroform and toluene, respectively;<br>• These sensing materials can be used in E-nose applications of detecting diseases (e.g., lung cancer) from EHB such that 80-100 ppb levels (approximate toluene concentration in lung cancer patient breath) is possible to detect with these sensors by pre-concentrating the VOCs in breath samples.
Benzene, Toluene, Isopropanol, Ethanol, Butanol, Acetone	[117] Shao (2017)	PtOx/GQDs/TiO2 nanoporous thin film	Sensor device with PtOx/GQDs/TiO2 nanoporous thin film, synthesized in situ using water vapor hydrothermal treatment and oxygenplasma treatment	Fabricate a tunable and sensitive VOCs sensor may lead to a potential real-world application for breath diagnosis	• The PtOx/GQDs/TiO2 nanocomposite thin films depict reversible change of sensing behaviour from p-type to n-type sensing behaviour for oxygen-functionalized VOCs as function of oxygen-plasma treatment, while these gas sensors present p-type sensing performance for aromatic VOCs throughout;<br>• These gas sensors exhibit high sensitivity to isopropanol gas with response value up to 4.4 to 1 ppm and a short response time of 9 second at room temperature.

Acetone, Ammonia, Carbon monoxide, Carbon dioxide	[114] Pace (2012)	Four MOX sensors (TGS-822, TGS-826, TGS-2600, TGS-2602) and an electrolyte sensor (TGS-4161), a temperature sensor (LM35), a relative humidity sensor (HIH4000) and a pressure sensor (XFAM115)	E-Nose system with Commercial Off-The-Shelf (COTS) sensors with different selectivity patterns	Implemented electronic nose systems have been effectively applied to gases mixtures detection in refinery environment and in monitoring of patients' breath on haemodialysis treatment	• The sensors response patterns changed during the treatment (about 4 h), in particular, sensor S1, specific for ammonia, showed a continuous decrease of its voltage response with dialysis time.
Ethanol, 2-propanol and n-butanol	[118] Hoppe (2018)	(CuO-Cu2O)ZnOAl heterojunctions	Chemical sensors	Detection of VOCs from human breath	• Detection of 100 ppm of different VOC vapors (ethanol, 2-propanol and n-butanol) • The (CuO-Cu2O)/ZnO:Al heterojunction showed a reduced dependence of the sensing properties with respect to relative humidity, which is very important for ambient gas sensing applications and VOC vapor detection in human breath analysis, chemical industry, in outdoor and indoor air quality monitoring.
Toluene, ethanol and 2,6-diisopropylphenol	[120] Seo (2011)	Porous films made of Au-loaded TiO_2 nanotubes	Resistive -sensor devices using the Au-loaded TiO2nanotubes	Detection of toluene, ethanol and 2,6-diisopropylphenol (a hypnotic drug for anaesthesia) at low ppm concentrations	• Promising applicability of the device for human breath analysis; • High sensor responses to large-sized VOC molecules; • The Au-loaded TiO2 nanotubes would offer a way to continuously monitor a change in the concentration of VOCs in exhaled air for medical diagnostics.

Toluene	[121] Suematsu (2018)	Pd-SnO_2-clustered nanoparticles	Pulse-driven resistive-type semiconductor gas sensors: heater-switching, pulse-driven, micro gas sensor composed of a microheater and a sensor electrode fabricated with Pd-SnO2-clustered nanoparticles as the sensing material	Responses toward toluene at levels of a few ppb	• The combination of a pulse-driven microheater and a suitable material designed to detect toluene resulted in improved sensor response, and facilitated ppt-level toluene detection; • It is able to screen for lung cancer as a cost-effective and compact human-breath analyser.
1,4-dimethoxy-2,3-butanediol, Sacyclohexanone, acetone, ethanol, methanol	[123] Sampson (2018)	Developed Quartz Tuning Forks (QTFs) modified with polystyrene and polymethylmethacrylate films	Quartz Tuning Fork(QTF) based sensors	Detection of low vapour pressure VOCs. QTF-PS film sensors were found to be responsive to cyclohexanone vapor, while QTF-PMMA wire sensors have been found to be good detectors of 1,4-dimethoxy-2,3-butanediol	• Measurement in an array of sensors with additional sensor having response towards particular VOCs. • These sensors maybe deployed in the field as a non-invasive preliminary tool to detect various bio markers of breast cancer in the exhaled breath of potential patients.
Benzene, toluene, xylene, acetone, ethanol and Formaldehyde	[122] Zhou (2018)	Synergetic p+n field effect transistor (FET) amplification circuit	n-type field effect transistor (n-FET) amplification circuit to amplify the voltage signal of MOX sensors and p-FET analogue amplification circuit is developed. p+n FETs are further synergistically integrated into an amplification circuit to increase the voltage signal of MOX sensors taking commercial sensor	ppb-level xylene detection; good selectivity over formaldehyde	• The ppb-level xylene detection for air quality monitoring and breath analysis using metal oxide semiconductor (MOX) gas sensors is successfully completed by proposed synergetic p+n amplification circuits; • Synergetic p+n amplification circuits can not only improve the sensor's response to different concentrations of xylene, but also decrease the detection limit of TGS2602 sensor to ~10 ppb xylene; • The sensor shows also good selectivity over formaldehyde, one typical gas exhaled by breast cancers and relative humidity existing in breath.

VOCs	[95] Longo (2018a)	Commercial MOX sensor (MiCS-5521)	GC/sensor system with the GC capillary column inserted by a splitter into a tiny chamber hosting a VOC sensor	Detection of VOCs in human semen samples	• Using exclusively sensor resistance profiles, accuracy is of 77% (in 2D) and 78% (in 3D) for discriminated human semen samples with different spermatic motility;<br>• VOC sensor has different affinity to organic compound classes, having a higher reactivity with aldehydes (72% of total VOCs), ketones and acetamides (50%)
VOCs	[97] Longo (2019)	Commercial MOX sensors	4-sensor array based on microm-achined gas sensors using (MOX) sensing elements	VOC detection in blood, urine, hair and human semen samples from young men living in an Italian pollutant area	• The response to hair sample has a different trend, since it's a drier matrix and consumes standard solvent, step by step, at single cycle;<br>• VOC composition in seminal plasma is much more abundant.
